# Metal-enriched HSP90 nanoinhibitor overcomes heat resistance in hyperthermic intraperitoneal chemotherapy used for peritoneal metastases

**DOI:** 10.1186/s12943-023-01790-2

**Published:** 2023-06-14

**Authors:** Qiang Wang, Peng Liu, Yingfei Wen, Kuan Li, Bo Bi, Bin-bin Li, Miaojuan Qiu, Shiqiang Zhang, You Li, Jia Li, Hengxing Chen, Yuan Yin, Leli Zeng, Changhua Zhang, Yulong He, Jing Zhao

**Affiliations:** 1 Digestive Diseases Center, The Seventh Affiliated Hospital of Sun Yat-Sen University, Sun Yat-Sen University, Shenzhen, Guangdong China; 2 Guangdong Provincial Key Laboratory of Digestive Cancer Research, The Seventh Affiliated Hospital of Sun Yat-Sen University, No. 628 Zhenyuan Road, Shenzhen, 518107 Guangdong China; 3 Scientific Research Center, The Seventh Affiliated Hospital of Sun Yat-Sen University, Shenzhen, Guangdong China; 4 Gastric Cancer Center, West China Hospital, and State Key Laboratory of Biotherapy, Sichuan University, Sichuan, China

**Keywords:** Heat shock protein 90 inhibitor, Epigallocatechin gallate, Heat stress resistance, Pyroptosis, Hyperthermic intraperitoneal chemotherapy

## Abstract

Clinical hyperthermic intraperitoneal chemotherapy (HIPEC) is regarded as a potential treatment that can prolong survival of patients with peritoneal metastases after cytoreductive surgery. However, treated tumor cells are prone to becoming heat resistant to HIPEC therapy through high expression of heat shock proteins (HSPs). Here, a carrier-free bifunctional nanoinhibitor was developed for HIPEC therapy in the management of peritoneal metastases. Self-assembly of the nanoinhibitor was formed by mixing Mn ion and epigallocatechin gallate (EGCG) in a controllable manner. Such nanoinhibitor directly inhibited HSP90 and impaired the HSP90 chaperone cycle by reduced intracellular ATP level. Additionally, heat and Mn ion synergistically induced oxidative stress and expression of caspase 1, which activated GSDMD by proteolysis and caused pyroptosis in tumor cells, triggering immunogenic inflammatory cell death and induced maturation of dendritic cells through the release of tumor antigens. This strategy to inhibit heat resistance in HIPEC presented an unprecedented paradigm for converting “cold” tumors into “hot” ones, thus significantly eradicating disseminated tumors located deep in the abdominal cavity and stimulating immune response in peritoneal metastases of a mouse model. Collectively, the nanoinhibitor effectively induced pyroptosis of colon tumor cells under heat conditions by inhibiting heat stress resistance and increasing oxidative stress, which may provide a new strategy for treatment of colorectal peritoneal metastases.

**Supplementary Information:**

The online version contains supplementary material available at 10.1186/s12943-023-01790-2.

## Introduction

Peritoneal metastases (PM), the common metastases originating from tumors in the abdominal and pelvic cavity, is associated with debilitating symptoms, clinical deterioration, and limited treatment opportunities. Colorectal cancer (CRC) is the third most frequently diagnosed cancer and second leading cause of cancer-related death worldwide [1]. Approximately 20% of patients with CRC develop peritoneal metastases [2, 3], and the median survival ranges from 5 to 7 months if untreated [4]. Apart from typical treatments involving surgery, intravenous systematic chemotherapy, and supportive care, a combined strategy of hyperthermic intraperitoneal chemotherapy (HIPEC) has provided effective treatment in some patients with peritoneal metastatic diseases [5].

In peritoneal metastases of colorectal origin, platinum agents and mitomycin C are used the most widely. Compared to systemic intravenous chemotherapy, HIPEC has several therapeutic advantages including favorable pharmacokinetics and homogeneity of chemotherapy and heat. Intraperitoneal delivery achieves about 20-fold greater drug concentrations at the peritoneal surface than in the plasma due to the blood-peritoneal barrier [6, 7]; moreover, the peritoneal clearance is much slower compared to systemic drug clearance, thus facilitating prolonged exposure of tumor cells to chemotherapy [8, 9]. In addition, heat enhances tissue penetration and cell membrane permeability, which results in better drug uptake in deep tumor tissues [6, 10]. These pharmacokinetic advantages increase the locoregional therapeutic effect while reducing systemic toxic effects.

The rationale of hyperthermia is based upon a direct thermal cell-killing effect on tumor cells, but not normal cells, when temperatures exceed 41–42 °C [11]. However, the optimal combination of HIPEC therapy has shown limited effectiveness, due to heat/drug resistance, thus hampering the application of HIPEC treatment [12, 13]. When cells are exposed to stress conditions including elevated temperature, heavy metals, chemical agents, etc., the heat shock proteins (HSPs) are synthesized in response. These stress-induced chaperones bind to client proteins to avoid protein misfolding and aggregation and protect cells from stress conditions. HSPs are generally categorized into six major families according to molecular size: HSP100, HSP90, HSP70, HSP60, HSP40, and small heat shock proteins [14, 15]. Among them, HSP90, widely expressed in many malignancies, has been reported as a promising target for tumor treatment [16–18]. Furthermore, many studies have reported HIPEC can induce expression of HSP90 [13, 19, 20]. The markedly increased level of HSP90 under HIPEC treatment provides a sufficient therapeutic window for tumor treatment. In this study, we aimed to create an agent that selectively targets tumor cells and makes them more vulnerable to heat and chemotherapy by inhibition of HSP90.

The HSP90 family members in mammals are known as HSP90α (in cytoplasm), HSP90β (in cytoplasm), glucose-regulated protein 94 (in endoplasmic reticulum), and the tumor necrosis factor receptor-associated protein 1 (in mitochondria). HSP90α is an inducible defense upon environmental or patho-physiological stress conditions [21, 22]. Structurally, HSP90α is a homodimeric protein, and each monomer is composed of three domains: N-terminal domain (NTD) for adenosine triphosphate (ATP) binding; the middle domain for client protein binding, and the carboxy-terminal domain (CTD) for HSP90 dimerization [23]. Currently, most existing drugs involved in clinical trials are NTD inhibitors, whereby the binding between NTD inhibitors and HSP90 triggers the heat shock response (HSR). The HSR is mediated by heat shock factor 1, resulting in compensatory expression of other heat shock proteins, such as HSP70, HSP40, and HSP27 [24, 25]. However, CTD HSP90 inhibitors might avoid such drawbacks. Therefore, the identification, characterization, and development of C-terminal inhibitors are recognized as a hot topic [26, 27].

Epigallocatechin gallate (EGCG), a highly bioactive natural constituent, has shown antitumor activities by binding directly to cellular and molecular receptors and signaling molecules, or by inhibiting the functions of key proteinases, kinases, and other enzymes. As a natural compound, EGCG has been considered clinically safe with low production cost [28, 29]. EGCG has entered phase-I or II clinical trials in treatment of colorectal, prostate, lung, breast, esophageal, bladder, pancreatic (NCT02336087), superficial skin (NCT02029352), and urothelial (NCT01993966) tumors, indicating its translational potential for cancer treatment [29, 30]. Yet the underlying mechanism is not fully understood.

In recent years, EGCG has also emerged as a promising HSP inhibitor, representing an attractive natural constituent for further development of a cancer therapy [31, 32]. However some intrinsic deficiencies restrict its application, such as structural instability and low bioavailability [30, 33]. Several investigators have made attempts to encapsulate EGCG in nano-sized vehicles to overcome these shortcomings in basic research [34–36].

Here, we developed a novel metal enriched HSP90 nanoinhibitor for hyperthermic intraperitoneal chemotherapy for the management of colorectal peritoneal metastases. The nanoscale HSP90 inhibitor was formed by combining EGCG and magnesium (Mn) using a flash nanocomplexation (FNC) technique. This HSP90 nanoinhibitor inhibited the biological function of HSP90 in tumor cells and made them more susceptible to heat stress. Moreover, the nanoinhibitor combined with heat induced pyroptosis of tumor cells by the caspase 1/GSDMD pathway. In a Balb/c mouse model with CT26 cells, HIPEC with the nanoinhibitor revealed tumor-targeting ability and long-term potent therapeutic effect with limited toxicity. HSP90 inhibition and antitumor effect were also observed in patient-derived tumor organoids. Overall, these data suggest that nanoinhibitor based HIPEC might be a promising therapeutic approach for treatment of colorectal peritoneal metastases.

## Methods

### Cell culture

The human colon adenocarcinoma cell line HCT116, human gastric mucosal epithelial cells line GSE1, and human peritoneal mesenchymal cell line HMrSV5 were purchased from the American Type Culture Collection (ATCC, USA). The human gastric cancer cell lines MGC803, HGC-27, and mouse colon adenocarcinoma cell lines CT26 and CT 26-lucifer were obtained from Procell Life Science & Technology Co., Ltd. (China). HCT116 cells were cultured in McCoy’s 5A medium with 10% bovine serum (Gibco). GSE1, HMrSV5, MGC803, HGC-27, CT26, and CT 26-lucifer cells were cultured in 1640 medium with 10% bovine serum (Gibco).All cells were incubated at 37 °C in a 5% CO_2_ humidified incubator.

### Fabrication and characterization of nanoihibitor

EGCG (45.8 mg) was dissolved in 10 mL water and the pH was adjusted to 7.5 with NaOH. MnCl_2_•4H_2_O (197.0 mg) was dissolved in 10 mL water and the pH was adjusted to 6.0 with Hcl. The MnEGCG nanoparticles were synthesized by a T-type confined impingement jet mixer (CIJM) with a flow rate of 5 mL/min. After centrifugation and wash with water, MnEGCG nanoparticles were obtained and kept in ethanol at -20 °C for subsequent analyses. The average particle size and zeta potential (ζ-potential) were measured using the laser light scattering Zetasizer (Nano ZS, Malvern) in water. X-ray diffraction (XRD) was obtained by Bruker D8 Advance. Fourier transform infrared spectroscopy (FTIR) spectra was evaluated on a Thermo Scientific Nicolet iS5 spectrometer in the range of 4000–400 cm^−1^. Mn content was confirmed by energy-dispersive X-ray spectroscopy (XPS, Thermo Scientific K-Alpha).

### In vitro cell uptake and cytotoxicity of nanoinhibitor

To evaluate the antiproliferation effect, cell viability was quantified in human tumor cells and mouse tumor cells using the cell counting kit-8 (CCK-8) and acetoxymethyl ester (AM)/propidium iodide (PI) cytotoxicity assay kit. The nanoinhibitor was added to the medium of cells plated in 96-well plates and incubated at 37 °C and 43 °C for 30 min, respectively, and then the medium was refreshed. The CCK-8 assay and AM/PI assay tests were conducted at 24 and 48 h. The modified formula was used to calculate cell viabilities, where cell viability % = (OD_sample_—OD_blank_ / OD_37℃ control_—OD_blank_) × 100%.

5-FAM cadaverine (0.5 μg/mL) and nanoinhibitor were mixed in ethanol and stirred for 24 h. CT 26 cells were treated with the labeled nanoinhibitor and free 5-FAM cadaverine, respectively. After incubation for 0, 4, 6, 9, and 12 h, the cells were washed twice with PBS and fixed with 4% paraformaldehyde, and then stained by DAPI. Fluorescence images were recorded by confocal laser scanning microscopy (CLSM, Zeiss LSM880, Germany).

### Western blot

CT26 cells were treated with PBS 37 °C, EGCG 37 °C, nanoinhibitor 37 °C, PBS 43 °C, EGCG 43 °C, and nanoinhibitor 43 °C. Protein samples were collected and purified from the lysates and supernatants of cells treated for 1, 6, and 24 h. HSP90α antibody (catalog AF1378), HSP70 antibody (AH728), β-actin antibody (catalog AF0003), calreticulin antibody (catalog AF16666), and HMGB1 antibody (catalog AF08180), were purchased from Beyotime Biological Co., Ltd. Caspase 1 antibody (catalog 22915–1-AP) and GSDMD antibody (catalog 66387–1-lg) were purchased from Proteintech Group, Inc. All protein samples were visualized and analyzed by Western blotting with appropriate antibodies.

### Molecular docking

Autodock Vina 1.2.3 [37] was used to dock the monomer and dimer forms of Hsp90 (PDB ID: 7L7J) [38] with small molecules EGCG globally. Before docking, hydrogen atoms were added to protein at pH = 7. Gaussian 16 was used to optimize the geometry of EGCG at the level of B3LYP/def-TZVP, and Multiwfn [39] was used to fit the RESP charge. According to the docking results, the docking sites and conformations with the highest binding affinity scores were selected for discussion, and the 3D and 2D images of receptor-ligand interactions were displayed using UCSF ChimeraX [40] and Discovery Studio 2021 [41].

### RNA extraction and sequencing

After CT26 cells were treated with nanoinhibitor (50 mg/L) and heat for 24 h, the biological samples (*n* = 3) were collected for RNA extraction by the TRIzol reagent kit (Invitrogen, USA). RNA sequencing and library construction were performed using the Illumina HiseqTM 2500/4000 by Gene Denovo Biotechnology Co., Ltd (Guangzhou, China). Gene expression profiles were analyzed by DESeq2 software. Enrichment pathway analyses, such as GO, KEGG, and GES (Gene Set Enrichment), were performed using Omicsmart, a real-time interactive online platform for data analysis.

### ATP and ROS assessment

After different treatments, the cells lysates and supernatants were collected. The intracellular and extracellular ATP levels were measured by a microplate reader (BioTek, SynergyH1, USA) after 3 min incubation with a standard ATP assay kit.

The treated cells were incubated with serum free DCFH-DA probe (10 µM) for 30 min at 37 °C under light exclusion. The level of reactive oxygen species (ROS) was determined by a fluorescence microscope (Leica, DMi8, Germany).

### Induction of immune response in vitro

#### Detection of pyroptosis

To capture pyroptotic cell morphology, the bright-field images were photographed using a LEICA DMi1 microscope. The supernatants were used to test lactate dehydrogenase (LDH) and IL-1β levels by the LDH Assay Kit and IL-1β ELISA Kit, respectively. The lysates of cells were used to quantify GSDMD, caspase 1, calreticulin (CRT), and high mobility group box 1 (HMGB 1) by Western blot as described previously [42]. The HMGB1 level in supernatants was also shown by Western blotting.

#### Activation and maturation of dendritic cells

Bone marrow derived cells (BMDC) were obtained from the femurs of Balb/c mice and cultured for 7 days to generate CD11c^+^ dendritic cells (DCs). CT26 tumor cells were cultured and treated in the upper well of a 24-well transwell system with 0.4 μm polycarbonate porous membranes for 24 h. The treated tumor cells were then incubated with the BMDCs seeded in the bottom well at a 2:1 ratio for another 24 h. The BMDCs were collected and stained with FITC CD11c antibody (catalog 117306), PE CD86 antibody (catalog 105007), and APC CD80 antibody (catalog 101713) and analyzed by flow cytometry. These antibodies were purchased from BioLegend, Inc.

### Animal studies

#### Mouse model for colorectal peritoneal metastases

The female Balb/c (4–5 weeks) mice were purchased from Charles River Co. Ltd. (Shanghai, China). All animal experiments in this study were in accordance with the National Regulation of China for Care and Use of Laboratory Animals approved by the Institutional Animal Care and Use Committee, Sun Yat-Sen University (SYSU-IACUC-2022-B1716). The mouse model for peritoneal metastases was induced 5 days after intraperitoneal injection of 2 × 10^6^ CT 26-lucifer cells per mouse. All mice were sacrificed via euthanasia method.

#### Biodistributions in vivo

To investigate the distribution of the nanoinhibitor in peritoneal metastases, the free DiR and nanoinhibitor conjugated with DiR were injected into two groups of tumor- bearing mice (*n* = 6). All mice were anaesthetized and photographed at 1, 6, 24, 48, 72, and 168 h by an in vivo imaging system (IVIS). Half of the mice were sacrificed and dissected at 72 h and 168 h after injection. The tumors and major organs were collected and imaged using IVIS. The fluorescence intensity was calculated using AnitView100.

#### Procedures of hyperthermic intraperitoneal chemotherapy

A total of 40 mice were divided into four groups, including control (PBS 37 °C), nanoinhibitor 37 °C, PBS 43 °C and nanoinhibitor 43 °C groups. The equipment for intraperitoneal perfusion mainly consisted of a heat exchanger, a roller pump, and a silicone tube. Mice were kept under isoflurane anesthesia (5%, 10 μL/g body weight), and their abdomen was sterilized with 75% alcohol. A 0.5-mm needle was inserted into the left upper abdomen as an inflow tube and a 0.9-mm needle was inserted into the right inferior abdomen as an outflow tube. The intraperitoneal temperature (higher than 41 °C but less than 43 °C) was constantly monitored by an infrared thermal imaging device and thermometer with a probe implanted in the inflow tube. The perfusate was pumped into the abdomen for 15 min (5 mL/min) until it reached the required temperature. To minimize damage and keep the mice alive, the procedure was adjusted or stopped once the liquid flow was blocked. Two cycles of HIPEC were performed on day 0 and day 7.

#### Antitumor effect in vivo

The experimental parameters were recorded every 5 or 10 days, such as body weights, abdominal circumference, and tumor growth. On day 17, half of the mice in each group were sacrificed, and the ascites and excised tumors were photographed and weighed. Tumors and main organs (heart, liver, spleen, lung, and kidney) of tumor-bearing mice after treatment were obtained for H&E and immunohistochemical analysis. The remaining mice were monitored until death for calculation of survival percentage. The tumor inhibition was calculated using the formula: (1-T/C) × 100% (T: mean weight of tumors in treatment groups, C: mean weight of tumors in control group).

Blood samples and ascites were collected for cytokine tests and biocompatibility assessment when the mice were sacrificed. IL-6, IFN-γ, and TNF-α were evaluated by a mouse IL-6 ELISA kit, mouse IFN-γ ELISA kit, and mouse TNF-α ELISA kit, respectively, according to the manufacturer’s protocol.

The freshly harvested tumors and major organs were fixed in 4% paraformaldehyde, dehydrated, embedded, and then sections were cut and placed onto slides. For histology, tissue slices were stained with hematoxylin and eosin. For immunohistochemistry, tissue slices were stained with CD3 (ABclonal, A19017), cleaved GSDMD (ABclonal, A22523), and HSP90 (Beyotime, AF1378) antibodies, respectively. Afterwards, stained slices were photographed with an optical microscope and analyzed.

Aspartate aminotransferase (AST), alanine aminotransferase (ALT), creatinine (CRE), and blood urea nitrogen (BUN) were tested for biosafety evaluation.

### Establishing organoids and evaluating the antitumor effect

At the beginning of the experiment, all patients provided informed consent. Following the guidelines of Helsinki Declaration and ethical regulations approved by Medical Ethics Committee of the Seventh Affiliated Hospital, Sun Yat-sen University (KY-2022–039-02), we obtained tumor biospecimens from two patients with colorectal peritoneal metastases between June 2022 and Septembers 2022. Those solid tumors were fragmented and seeded into 6- or 96-well plates by encapsulating cells (density of 2 × 10^5^ cells/mL) in a hydrogel. On the 5th day, the organoids were treated and then replenished with fresh medium and cultured for another 3 days, after which, experiments, such as cell viability, protein extraction, etc., were conducted.

### Statistics

The data are shown as mean ± standard deviation. GraphPad prism 8.0 software was used for the statistical analysis of the data. One-way ANOVA and Holm–Sidak test were used for multiple comparisons, T-tests were performed for comparisons of two groups, and the significant difference was showed at a value of *P < 0.05, **P < 0.01, ***P < 0.001, and ****P < 0.0001. *P* > 0.05 is indicated as not significant (ns).

## Results

### Synthesis and characterization of HSP90 nanoinhibitor

**Fig. 1:**
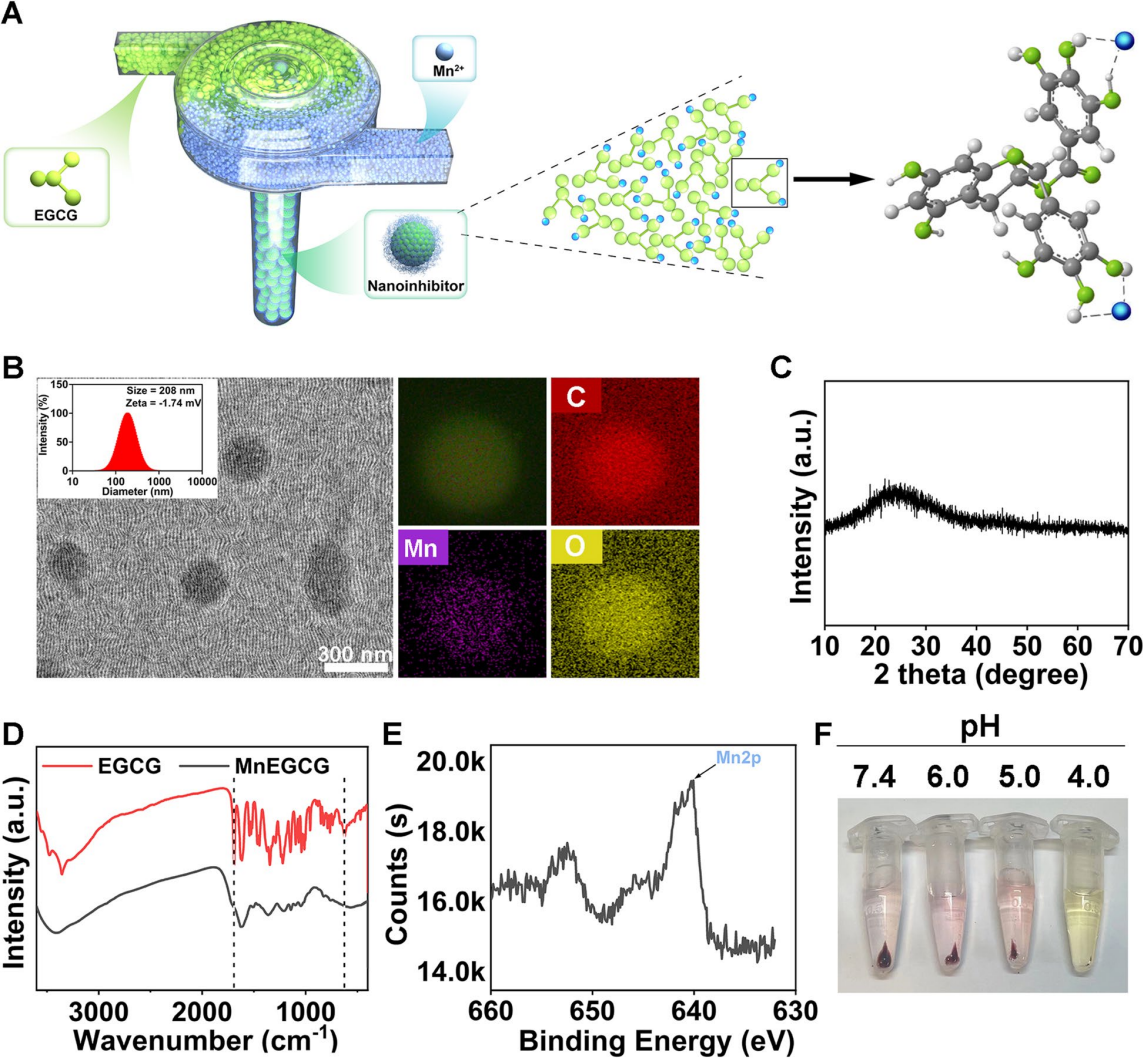
Design and characterization of MnEGCG nanoparticles. **A** Schematic illustration of the strategy for synthesis of MnEGCG nanoparticles. **B** TEM images of MnEGCG nanoparticles; the inset is the particle size distribution. C, Carbon. Mn, Manganese. O, Oxygen. **C** XRD, (**D**) FTIR and (**E**) XPS spectrum of MnEGCG nanoparticles. **F** Degradation of the nanoparticles in PBS at different pH values

Herein, MnEGCG nanoparticles were prepared by the flash nanocomplexation technique [43, 44]. As shown in Fig. 1A, EGCG and MnCl_2_•4H_2_O aqueous solutions were rapidly mixed by a two-stream confined impingement jet mixer (CIJM) with flow rate of 5 mL/min. As prepared, the nanoinhibitor was in amorphous spherical shape with average size of 208 nm (Fig. 1B and C). The nanoparticles had a negative surface charge with zeta potential around − 1.74 mV. The disappeared absorption peak of Fourier transform infrared spectroscopy (FTIR) centered at 1698 cm^−1^ of C-O and 623 cm^−1^ of phenolic hydroxyl group revealed the coordination of Mn^2+^ with phenolic hydroxyl groups on EGCG (Fig. 1D) [45, 46]. The Mn content was calculated to be 8.90 wt. % from X-ray photoelectron spectroscopy (Fig. 1E), and both Mn^2+^ and EGCG can be released from nanoinhibitor in acidic conditions, which is similar to the tumor microenvironment (pH 6.2 ~ 6.8) and cytolysosome (pH 4.5 ~ 5) (Fig. 1F).

### Cellular uptake and antiproliferation effect of the nanoinhibitor in vitro

**Fig. 2:**
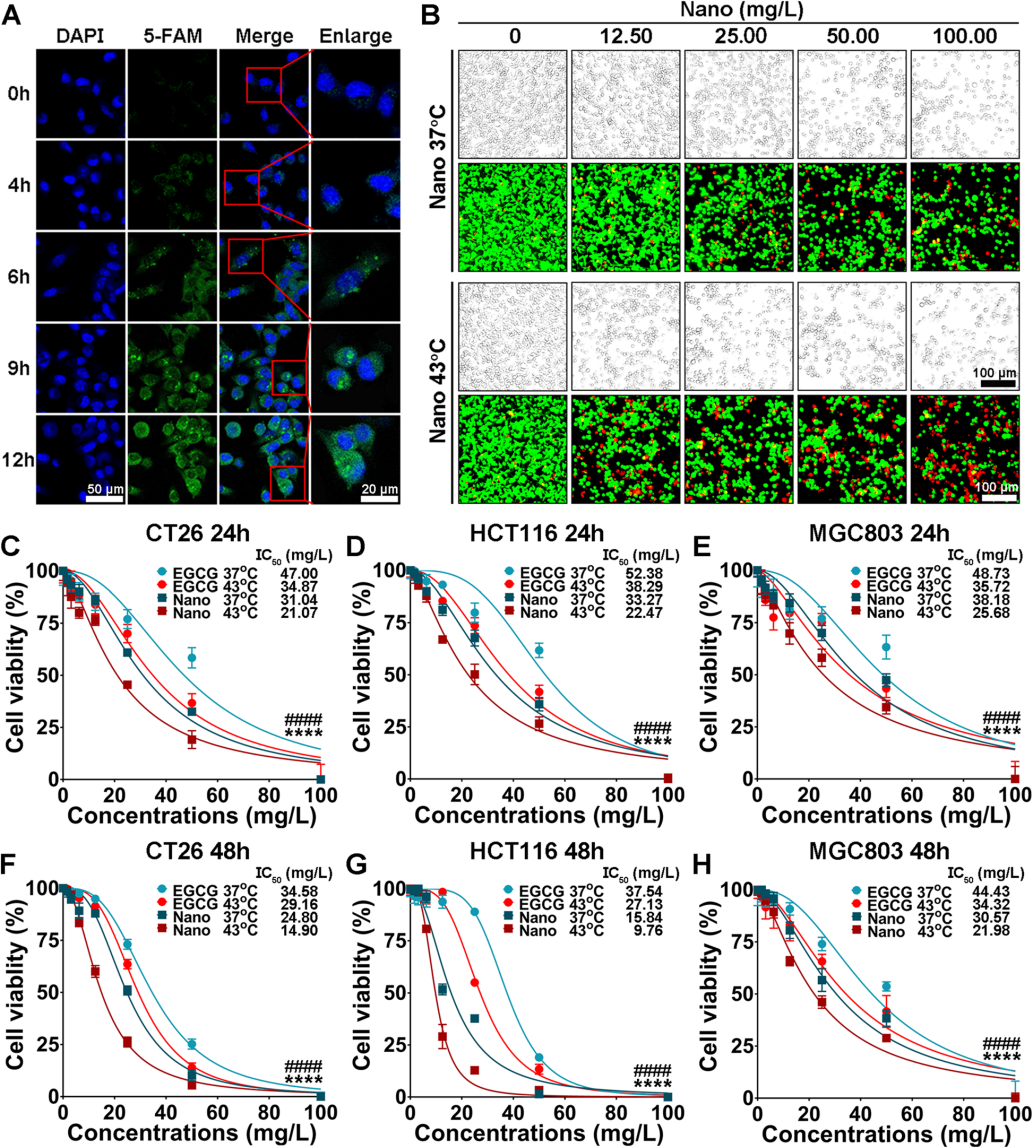
Cellular uptake and antiproliferation effect of nanoinhibitor in vitro. **A** Confocal fluorescent images showing cellular uptake of 5 FAM-nanoinhibitor in CT26 cells at 4, 6, 9, and 12 h. Green fluorescence represents 5-FAM conjugated to nanoinhibitor, and blue fluorescence indicates nucleus (DAPI). **B** AM/PI staining images of CT26 cells after treated with the nanoinhibitor at the concentrations of 12.50, 25.00, 50.00, and 100.00 mg/L for 24 h. Green fluorescence represents living cells dyed by acetoxymethyl ester (AM), and red fluorescence represents dead cells dyed by propidium iodide (PI). Cell viabilities after different treatments in CT26 cells for 24 h (**C**), HCT116 cells for 24 h (**D**), MGC803 cells for 24 h (**E**), CT26 cells for 48 h (**F**), HCT116 cells for 48 h (**G**), and MGC803 cells for 48 h (**H**). *, EGCG 37 °C vs EGCG 43 °C, ****p < 0.0001. #, Nano 37 °C vs Nano 43 °C, ####*p* < 0.0001. Nano, nanoinhibitor

The cellular uptake of nanoinhibitor was investigated by CLSM for 12 h. As shown in Fig. 2A, after the cells were incubated with the nanoinhibitor labelled with 5-FAM, a time-dependent cellular uptake was observed, and the fluorescence intensity inside the cells increased significantly at 6 h, thus confirming the successful uptake of the nanoinhibitor by tumor cells.

CCK-8 assays and AM/PI staining were used to evaluate the antiproliferation effect in vitro. Cell viability was detected when tumor cells were treated with EGCG 37 °C, nanoinhibitor 37 °C, EGCG 43 °C, and nanoinhibitor 43 °C for 24 or 48 h. At increasing concentrations, the nanoinhibitor resulted in significantly higher degree of tumor cell death (Fig. 2B). At equal concentrations, the nanoinhibitor displayed greater cytotoxicity than EGCG in mouse colon cancer CT26 cells, and heat further enhanced the antiproliferation effect (Fig. 2C, F). The IC_50_ of the nanoinhibitor plus 43 °C heating was 14.90 mg/L at 48 h, lower than that of 24 h (21.07 mg/L). A similar result was also observed in the HCT116 human colon cancer cells (Fig. 2D, G) and MGC803 human stomach cancer cells (Fig. 2E, H). However, soluble Mn^2+^ was not acceptable to inject directly into the body and showed limited cytotoxicity in cancer cells (Fig. S1). These results suggested the nanoinhibitor can significantly reduce resistance of tumor cells to heat treatment.

### Mechanism of antiproliferation

**Fig. 3:**
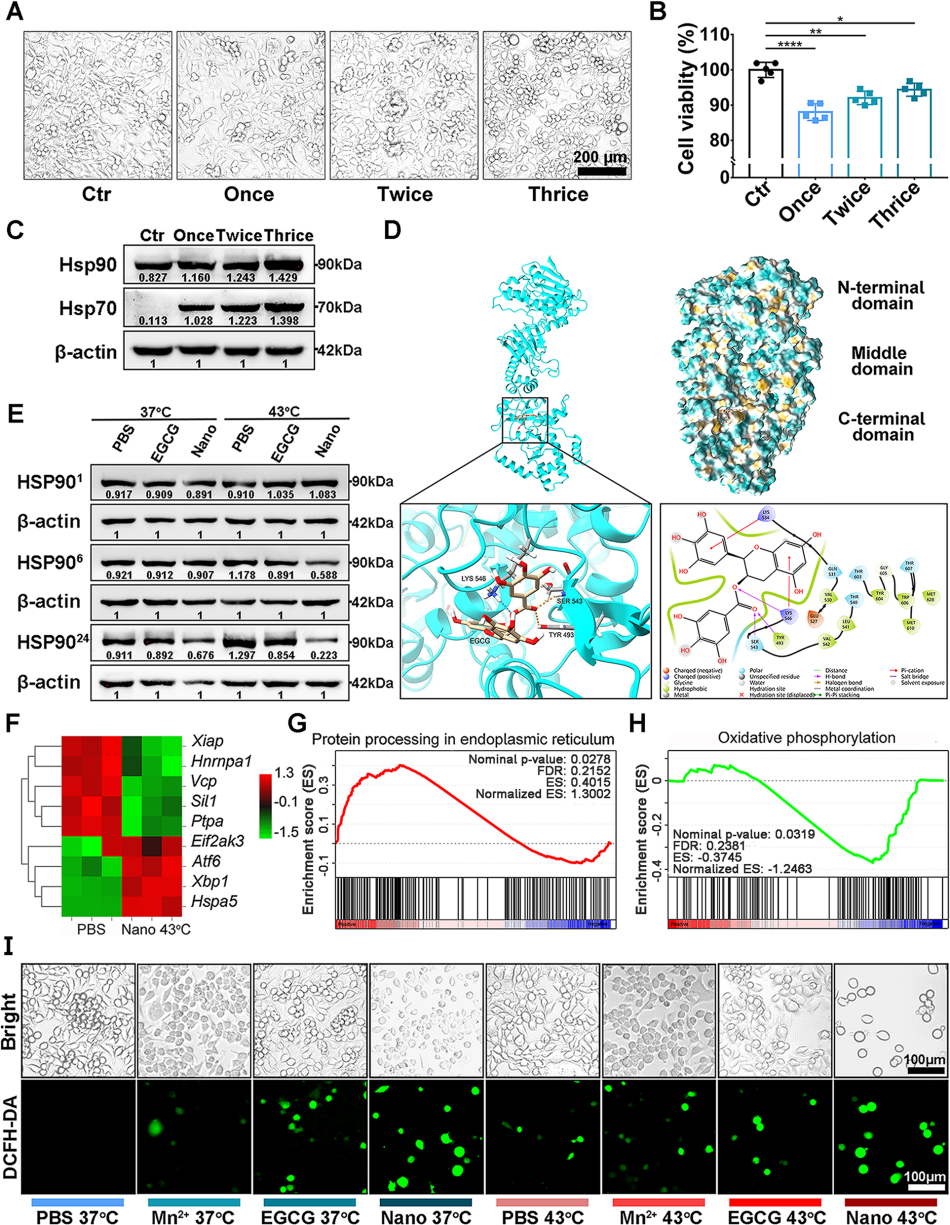
Antiproliferation mechanism of nanoinhibitor. **A** Brightfield images of tumor cells after treatment with the non-lethal high temperature (39 °C) once, twice, or thrice, respectively. Each heat session lasted for 30 min. **B** Cell viabilities after treatment with non-lethal high temperature (39 °C) corresponding to (**A**). **C** Western blot analysis of the effect of high temperature on the expression of heat shock proteins. **D** Docking models of EGCG bound to human HSP90α, and the structural view of EGCG in the HSP90 C-terminal domain. **E** Western blot analysis of HSP90 level in tumor cells treated with EGCG 37 °C (50 mg/L), nanoinhibitor 37 °C (50 mg/L), EGCG 43 °C (50 mg/L), or nanoinhibitor 43 °C (50 mg/L) at different time points. HSP90^1^, HSP90^6^, and HSP90.^24^, stand for HSP90 expression for 1, 6, 24 h after treatment, respectively. **F** Heat map of differentially expressed genes related to endoplasmic reticulum stress. **G**, **H** GSEA plots of differentially expressed genes enriched in (**G**) the protein processing in endoplasmic reticulum, and (H) oxidative phosphorylation. **I** ROS production after different treatments in CT26 tumor cells. *p < 0.05, **p < 0.01, and ****p < 0.0001

First, the antiproliferation effect of heat was investigated. After incubation at a non-fatal high temperature (39 °C) for 30 min, approximately 90% of tumor cells survived after 24 h, though the cell viabilities gradually increased with the rising number of heated times (Fig. 3A and B). Meanwhile, the expression of heat shock proteins (HSP90 and HSP70) increased (Fig. 3C). These results indicated the resistance of tumor cells could be induced by heat. To investigate the possible binding mode between EGCG and HSP90, we performed molecular docking. As shown in Fig. 3D, EGCG revealed a high binding activity with the C-terminal domain of the HSP90 homodimer, in accordance with binding site reported by other researchers (near the ATP-binding pocket, but not the ATP-binding pocket in the CTD) [47, 48]. After tumor cells were treated with heat, the expression of HSP90 increased in a time-dependent manner within 24 h. We analyzed HSP90 level at different time points up to 24 h. The HSP90 had almost no change at 1 h post-heat and started to regress at 6 h when the nanoparticle was gradually internalized into tumor cells (Fig. 3E). The inhibition of HSP90 was also observed in HCT116 cells after treatment (Fig. S2). RNA sequencing analysis was further conducted to assess the differentially expressed genes and enrichment pathways related to heat stress response. As shown in Fig. 3F, cell stress/death stimulatory genes, such as eukaryotic translation initiation factor 2 alpha kinase 3 (*Eif2ak3*), activating transcription factor 6 (*Atf6*), and X-box binding protein 1 (*Xbp1*), were up-regulated, and cell stress/death suppressor genes, such as X-linked inhibitor of apoptosis (*Xiap*), valosin containing protein (*Vcp*), and Sil1 nucleotide exchange factor (*Sil1*), were down-regulated after treatment with 43 °C heating and the nanoinhibitor. These genes were enriched in the protein processing in endoplasmic reticulum and oxidative phosphorylation (Fig. 3G, H).

HSP90 is an ATP-dependent molecular chaperone. In the absence of ATP, HSP90 is unable to regulate the structure and conformational cycle [49]. To further investigate the impact of the nanoinhibitor on HSP function, we evaluated the intracellular ATP level. As shown in Fig. S3, ATP levels decreased in EGCG and nanoinhibitor group after treatment, especially in the 43 °C nanoinhibitor treatment group. Thus the reduced intracellular ATP synergistically contributed to the inhibition of HSPs, and eventually enhanced the antiproliferation effect of EGCG and nanoinhibitor.

In addition, Mn ion (Fenton reaction inducer) and heat also synergistically contributed to the increased generation of ROS, which serves as an endogenous oxidative stress [50, 51]. The ROS level was evaluated at 24 h after treatment, and the group treated with the nanoinhibitor showed a higher ROS in comparison to EGCG, and a further increased level of ROS under heat. Overall, cells exposed to 43 °C heating with the nanoinhibitor showed the highest level of ROS (Figs. 3I and S4).

### Pyroptosis and immunostimulation induced by nanoinhibitor

**Fig. 4:**
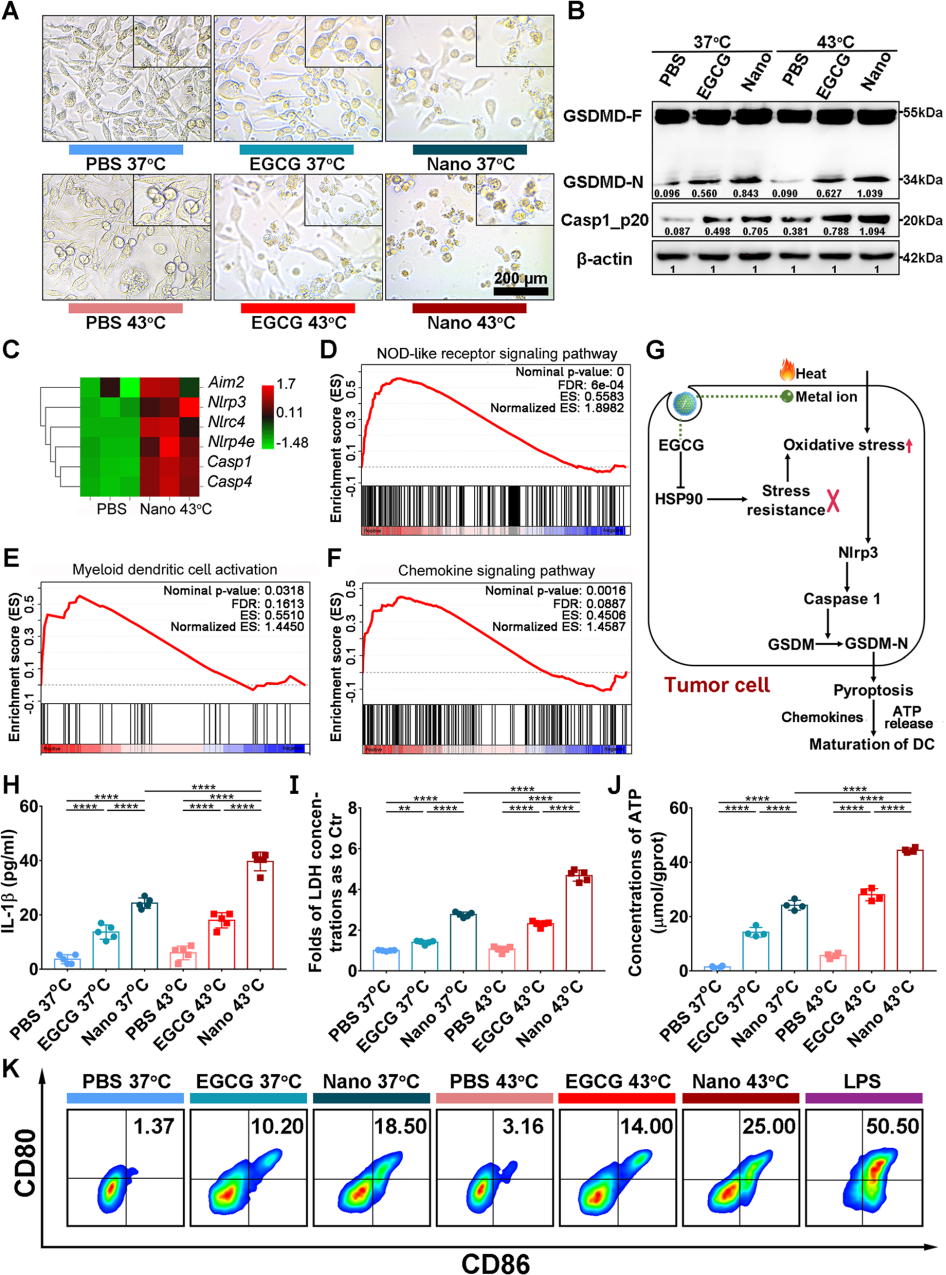
Tumor cell death and immunostimulation induced by nanoinhibitor in vitro. **A** Pyroptosis induced by different treatments. The top-right images are shown at twofold magnification. **B** Western blot analysis of pyroptosis-related proteins (the canonical pathway, GSDMD, Caspase 1) level. **C** Heat map showing differentially expressed genes related to pyroptosis. **D**-**F** GSEA plots of differentially expressed genes enriched in nucleotide oligomerization domain (NOD)-like receptor signaling pathway (**D**), myeloid dendritic cell activation (**E**), chemokine signaling pathway (**F**). **G** Illustration of pyroptosis by combined therapy of nanoinhibitor and 43 °C heating. EGCG, epigallocatechin gallate. HSPs, heat shock proteins. GSDM, gasdermin. DC, dendritic cell. **H**, **I** ELISA for IL-1β (**H**) and LDH (**I**) release of tumor cells after different treatments. **J** Extracellular ATP level of tumor cells after different treatment. **K** Flow cytometry of in vitro DCs maturation (CD80^+^CD86^+^, gated on CD11c.^+^) proportion (%) after incubation with CT26 cells with different treatments. Casp1_p20, cleaved caspase 1. LPS is regarded as the positive control group. LPS, Lipopolysaccharide. **p < 0.01, ****p < 0.0001

Excessive ROS is a reported effective inducer of pyroptosis in tumor therapy [52]. Pyroptosis, characterized by pore formation in membranes and cellular swelling with large bubbles and lysis, can trigger an immunogenic form of programmed cell death through the release of cellular contents. Pyroptosis is induced by canonical and noncanonical pathways mediated by the gasdermin protein family (GSDM, mainly GSDMD and GSDME). Gasdermin is cleaved by the caspase protein, producing a gasdermin-N domain fragment that inserts into cell membranes and thereby executes pyroptotic tumor cell death [53–55]. To better understand gasdermin’s key role in mediating nanoinhibitor-induced pyroptosis, we analyzed expression levels of GSDMD and GSDME in normal tissues and various tumor cell lines extracted from the *Expression Atlas* (https://www.ebi.ac.uk/gxa). The data indicated relatively higher expression of gasdermin protein in gastrointestinal organs compared to other organs (Fig. S5A and B), but reduced to different extents in tumor cell lines compared to normal tissues (Fig. S5C). We then observed the effect of the nanoinhibitor on tumor cells with different expression levels of gasdermin, where gasdermin-underexpressed HGC-27 cells developed non-pyroptotic morphology after treatment with the nanoinhibitor (Fig. S5D). Gasdermin-overexpressed CT26 cells showed evident cell swelling with typical giant bubbles from the plasma membrane (Fig. 4A), the same phenomenon was also observed in HCT116 cells after treatment with the nanoinhibitor (Fig. S6). Next, to elucidate the underlying mechanism, the pyroptosis-related proteins were investigated in CT26 cells. Caspase 1 activation was induced by the nanoinhibitor and amplified by heat. Subsequently, GSDMD was cleaved by caspase 1, and the levels of the GSDMD-N fragment were concurrently increased in both 37 °C and 43 °C nanoinhibitor groups, especially in the 43 °C nanoinhibitor group (Fig. 4B). The increased level of the GSDMD-N fragment was also observed in HCT116 cells treated with nanoinhibitor (Fig. S7). RNA sequencing analysis showed that pyroptosis-associated genes, including absent in melanoma 2 (*Aim2*), NLR family pyrin domain containing 3 (*Nlrp3*), and caspase 1 (*Casp1*), were up-regulated after treatment with nanoinhibitor and 43 °C heating (Fig. 4C). Related genes were enriched in the pyroptosis-associated pathway (Fig. 4D) and immune-associated pathway (Fig. 4E, F). The above results provided supporting evidence for elucidating the mechanism of pyroptosis after treatment with nanoinhibitor and heat (Fig. 4G). Metal ion and heat synergistically contributed to cell oxidative stress, which stimulated receptor patterns to assemble and activate the Nlrp3 inflammasome, the inflammasome can further activate the caspase 1 to induce the cleavage of gasdermin D. EGCG further increased oxidative stress by inhibiting heat stress resistance, consequently, tumor cells accumulated much cell stress that caused cell pyroptosis.

Further experiments were performed to verify the RNA sequencing analysis by chemokine testing and DC cell maturation assay. The leak of intracellular IL-1β, lactate dehydrogenase (LDH), and ATP outside the cell was also significantly higher in the nanoinhibitor-treated tumor cells undergoing pyroptosis (Fig. 4H-J). Taken together, the above results indicated that the nanoinhibitor induced pyroptosis through the caspase 1/GSDMD pathway in colon tumor cells and this effect can be augmented by heat.

Pyroptosis can induce the increase of CRT and the release of HMGB1 and ATP in tumor cells. Accumulating evidence suggested that such cellular contents can contribute to induce maturation of dendritic cells that trigger the adaptive immune response [56, 57]. The CRT protein was upregulated in the 43 °C nanoinhibitor group compared with other groups, and increased HMGB1 were released into the tumor microenvironment (Fig. S8).

After pre-treatment with the nanoinhibitor, the dying tumor cells were co-cultured with bone marrow derived cells for 24 h, and then antibody stained DCs were measured by flow cytometry. In similar total number of naive DCs in different groups (Fig. S9A), a significant increase in mature DCs was observed in the nanoinhibitor group, and combining 43 °C heating substantially improved the efficacy of the nanoinhibitor (Figs. 4K and S9B), suggesting the naive DCs can be activated by tumor antigens released from nanoinhibitor treated CT26 cells. These data indicated that the ICD caused by nanoinhibitor was capable of stimulating DCs maturation.

### In vivo biodistribution of nanoinhibitor

**Fig. 5:**
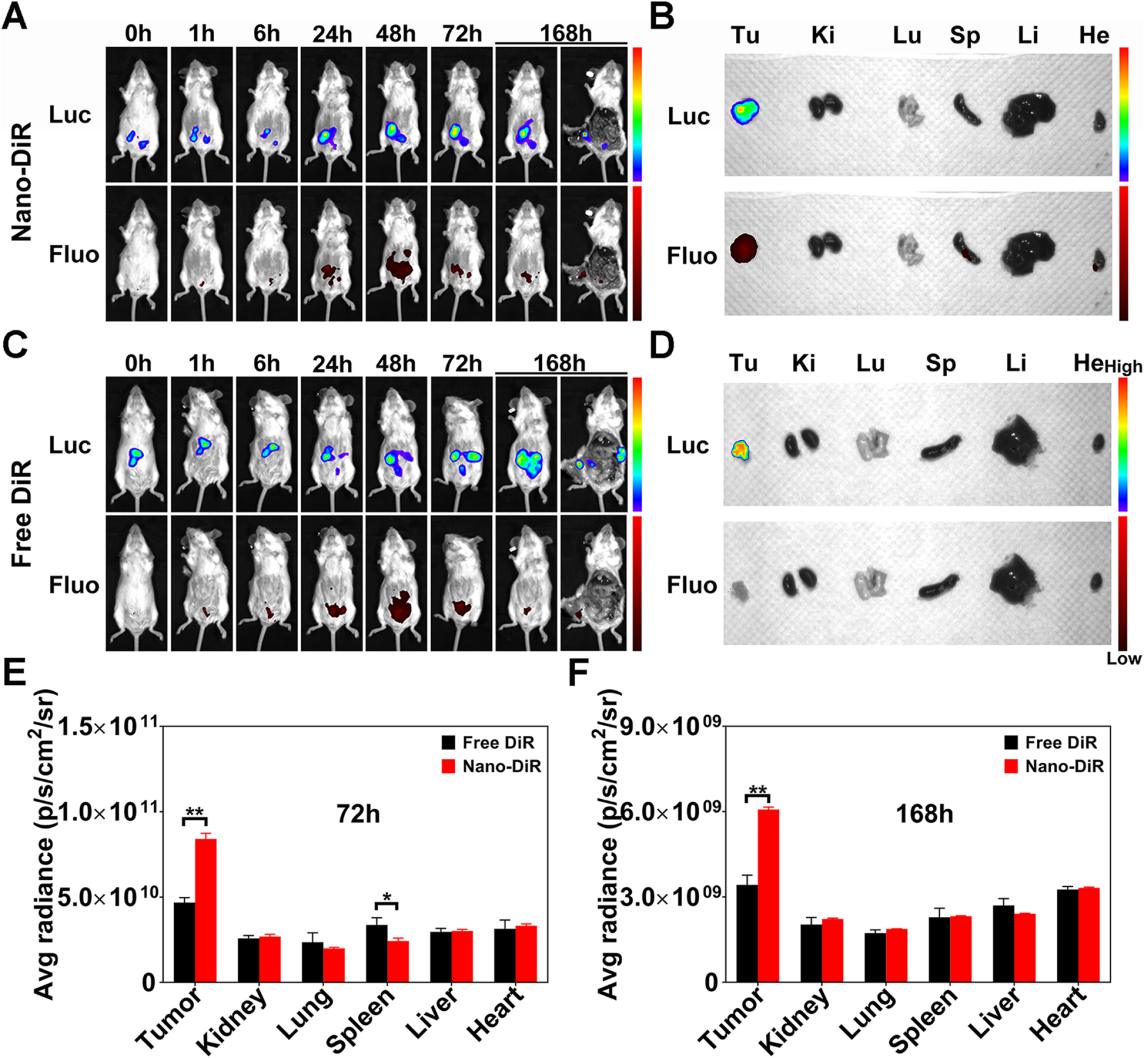
The biodistribution of the nanoinhibitor in tumor-bearing mice. Images of bioluminescence (Luc) for tumor and fluorescence (Fluo) for DiR in CT26-Luc tumor-bearing mice after intraperitoneal treatment with (**A**) nanoinhibitor-DiR, (**C**) free DiR at different time points. Images of bioluminescence (Luc) and fluorescence (Fluo) for tumors and main organs in (**B**) nanoinhibitor-DiR group, (**D**) free DiR group at 168 h intraperitoneal injection. **E**, **F** The quantitative fluorescence analyses of tumors and major organs in the two groups of mice at 72 h and 168 h intraperitoneal injection, respectively. Tu, tumor. Ki, kidney. Lu, lung. Sp, spleen. Li, liver. He, heart. Avg, average. *p < 0.05 and **p < 0.01

To study in vivo biodistribution and the antitumor effect, a syngeneic mouse model was established through transplantation of luciferase labeled CT26 into the abdominal cavity. Five days after tumor cells injection, the peritoneal metastases were confirmed by IVIS and in dissected tumors. Solid tumors of approximately 6 mm in diameter were found in the upper right quadrant of the peritoneum and under the left lobe of the liver. Several micro-tumor nodules (1–2 mm in diameter) were uniformly distributed in the mesentery (Fig. S10). The tumor-targeting ability was investigated by IVIS by intraperitoneally injecting free DiR and nanoinhibitor labeled with DiR. In the nanoinhibitor-DiR group, the tumor site was clearly visualized by the luciferase marker, and the gradual increase in fluorescence of the nanoinhibitor-DiR overlapped with the bioluminescence of the tumor, whereas free DiR distributed randomly throughout the peritoneal cavity within 48 h (Fig. 5A, C). Fluorescence from the labeled nanoinhibitor was also photographed in the tumors dissected at 72 h and 168 h after treatment (Fig. 5B, D-F). These results indicated that nanoinhibitor exhibited a long retention time and good tumor-targeting capacity in the abdominal cavity.

### In vivo antitumor evaluation of HIPEC

**Fig. 6:**
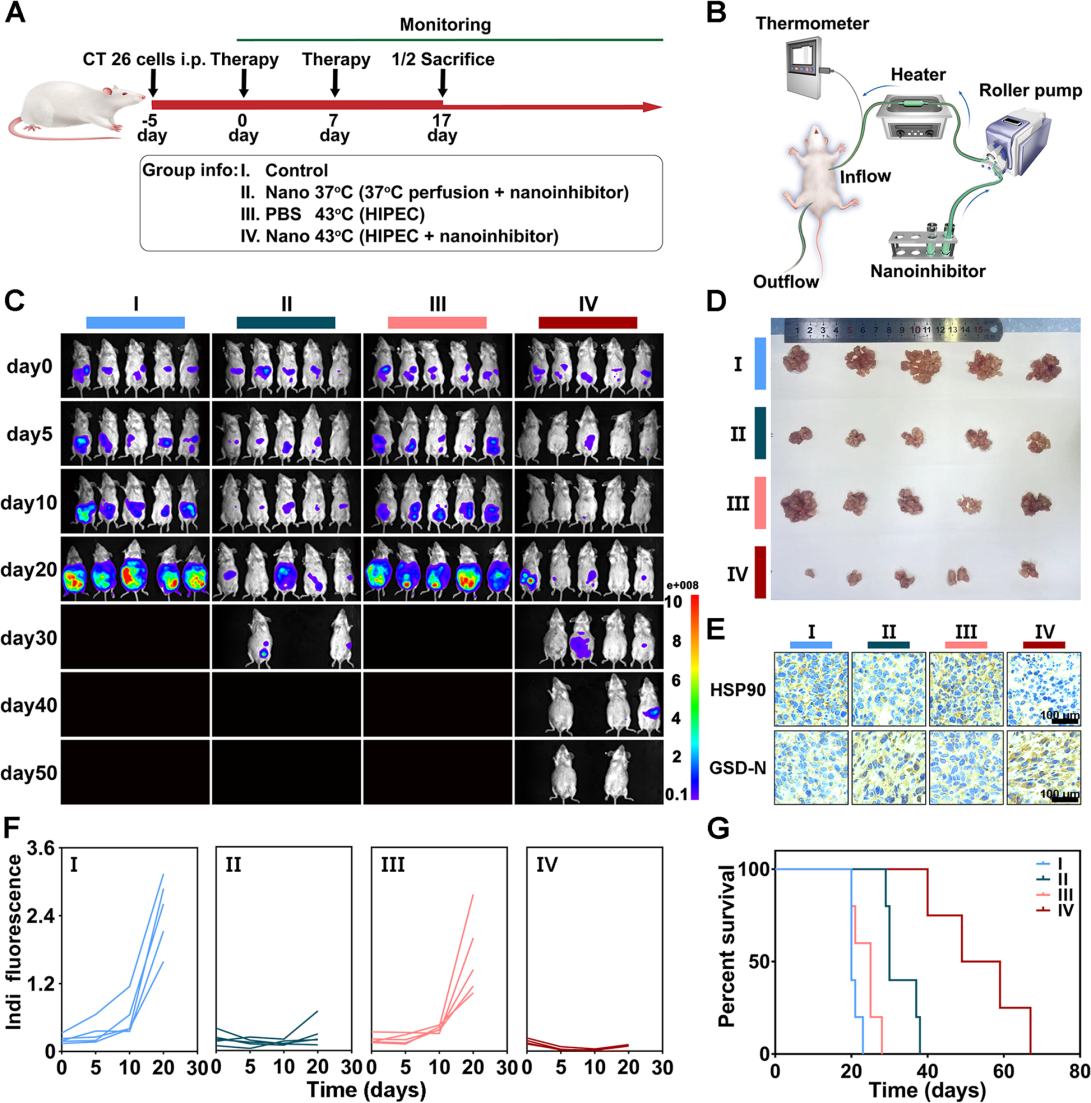
Antitumor effect of nanoinhibitor in vivo. **A** Schematic illustration of the experimental protocol in CT26-Luc tumor-bearing Balb/c mice. **B** Schematic illustration of the hyperthermic intraperitoneal perfusion procedure. **C** Bioluminescence images of tumor-bearing mice individually treated with PBS, Nano 37 °C (50 mg/L), PBS 43 °C, Nano 43 °C (50 mg/L) at day 0, 5, 10, 20, 30, 40, and 50. **D** Representative images of tumor tissues harvested from intraperitoneal at day 17. **E** Tumor immunohistochemical staining of HSP90 and cleaved GSDMD in different treatment groups. GSD-N, GSDMD-N fragment. **F** Bioluminescence intensity of tumors in different groups of mice individually corresponding to (**C**). **G** Survival curves of mice receiving the indicated treatment. i.p., intraperitoneal injection. Indi, individual

Encouraged by tumor cell inhibition and tumor targeting experiments, we assessed the antitumor performance of the nanoinhibitor with HIPEC therapy in the CT26-Luc tumor syngeneic mice model. With tumor cells injected intraperitoneally 5 days prior, mice were randomly divided into four groups (n = 10 per group) and received treatments with PBS, nanoinhibitor 37 °C (50 mg/L), PBS 43 °C, and nanoinhibitor 43 °C (50 mg/L). On day 17, five mice in each group were sacrificed to evaluate the physiological indicators and oncology parameters, and the rest of the mice were continuously monitored until the end of the experiment (Fig. 6A).

HIPEC was performed under a constant temperature of 41–43 °C (Figs. 6B and S11), as higher temperatures may lead to intraperitoneal injuries in mice. Following the strict disinfection protocol and thermal control during the HIPEC procedures, no hyperthermic perfusion related complications, such as intestinal adhesion and obstruction, were observed in any mice. The HIPEC procedure was stopped in the first mouse of the 43 °C nanoinhibitor group because of repeated outflow catheter obstruction, so this mouse was excluded when evaluating oncological parameters.

**Fig. 7:**
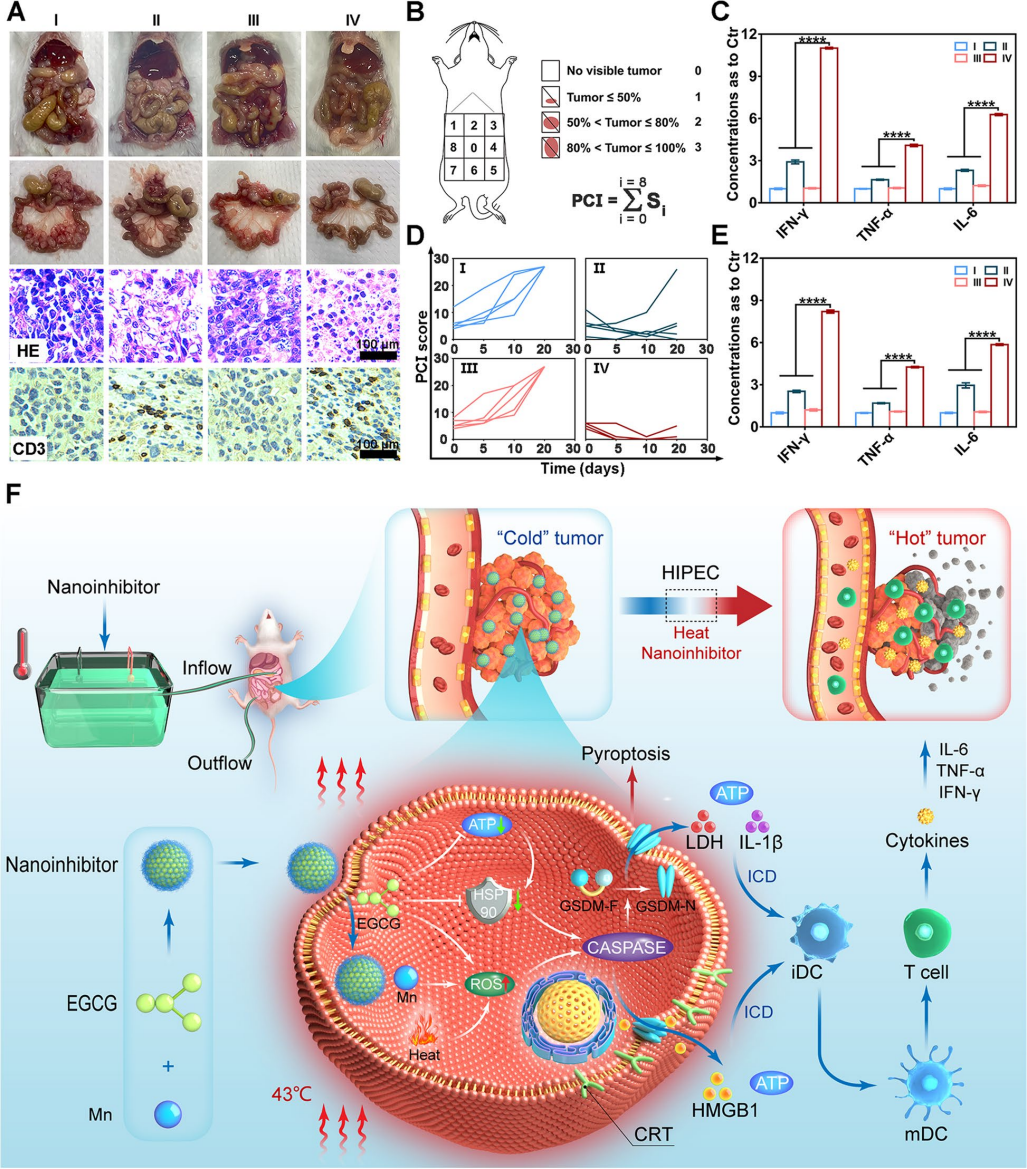
In vivo immune-stimulation induced by nanoinhibitor. **A** Representative photographs of abdominal cavity and mesenteries of mice after different treatments. H&E staining and CD3 immunostained images of tumors in different groups. **B** Schematic illustration of modified PCI score derived from human PCI score. **C** IFN-γ, TNF-α, and IL-6 concentrations in ascites compared with control group post-treatment. **D** Modified PCI score of mice individually in different groups. **E** IFN-γ, TNF-α, and IL-6 concentrations in serum compared with control group post-treatment. **F** Illustration of proposed antitumor mechanism of the nanoinhibitor. ****p < 0.0001

According to bioluminescence intensity using IVIS system, the nanoinhibitor showed significant inhibition of tumor growth, and its activity was further enhanced when HIPEC was combined (Figs. 6C, F and S12). Based on the tumor weights on the 17th day, mice in the control group exhibited rapid tumor growth; treatment groups inhibited tumor growth to a greater or lesser extent (Fig. 6D). Notably, tumor inhibition rate in the 37 °C nanoinhibitor group was calculated to be 43.5%, which was significantly inferior to 74.6% in the 43 °C nanoinhibitor group (Fig. S13). The effect of HSP90 inhibitory and pyroptosis simulation were also confirmed by immunohistochemical and western blot analysis (Figs. 6E and S14). The nanoinhibitor based HIPEC therapy significantly prolonged the survival of tumor-bearing mice, shown in Kaplan–Meier curves (Fig. 6G). Tumor dissemination in the abdominal cavity was also investigated, and different sizes of disseminated tumors spread over the abdominal cavity, especially in the intestine and mesentery in the control and 43 °C PBS groups. Several large tumors showed in the mesentery in 37 °C nanoinhibitor group, while a few small tumors were observed in the 43 °C nanoinhibitor group. The performance of nanoinhibitor and heat in tumor inhibition was further confirmed by H&E staining of tumor tissue (Fig. 7A). All results suggested an excellent chemo-hyperthermic synergistic effect between the nanoinhibitor and hyperthermic perfusion for disseminated tumors located even deep in the abdominal cavity. The nanoinhibitor played dominant roles in the multimodality treatment, which was consistent with the mainstream view that the therapeutic effect of HIPEC mainly relies on the chemotherapeutic agents [58].

The living tumor burden was assessed using the modified peritoneal carcinomatosis index (PCI) according to the bioluminescence images. Mouse abdomen was averaged into 9 regions, the PCI score was determined by summing the score of each region based on tumor size (Fig. 7B). Like the results of bioluminescence intensity, the mice showed the lowest PCI score in the 43 °C nanoinhibitor group (Figs. 7D and S15). Immune response was supposed to be involved in the antitumor process. In order to investigate biological effects of the combined treatments in tumor microenvironment, immunohistochemical analysis was performed to characterize the tumor content. Notably, tumors treated with nanoinhibitor-based HIPEC showed the highest level of CD3 (Figs. 7A and S16), which is a marker of T cell infiltration in the tumor microenvironment. In the study, significantly elevated levels of IFN-γ, TNF-α and IL-6 were observed in the ascites after treatment with nanoinhibitor-based HIPEC (Fig. 7C). Cytokines in blood circulation were further detected for evaluating systemic immune response, and treatment groups showed similar results (Fig. 7E). Thus, the therapy efficiently converted the “cold” tumors into “hot” tumors (with increased locoregional immune response and systemic immune response, Fig. 7F).

### Translational research of the nanoinhibitor in organoids

**Fig. 8:**
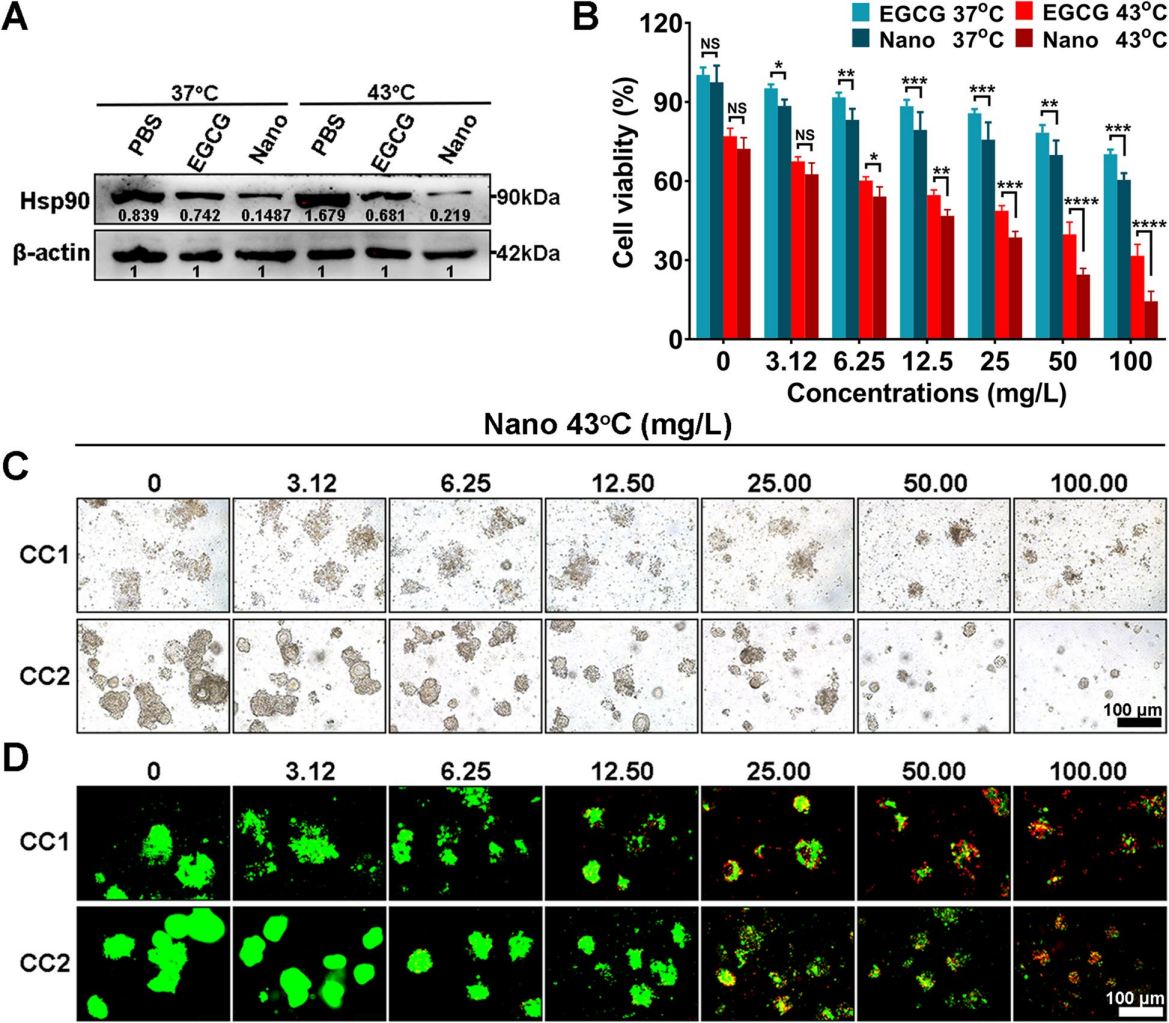
Translational research of nanoinhibitor in organoid. **A** Western blot analysis of heat shock protein 90 level in organoids from a CRC tumor patient after treatment with PBS, EGCG 37 °C (50 mg/L), Nano 37 °C (50 mg/L), PBS 43 °C, EGCG 43 °C (50 mg/L), Nano 43 °C (50 mg/L). **B** Cell viabilities of organoid after treatment with EGCG 37 °C, Nano 37 °C, EGCG 43 °C and Nano 43 °C at the drug concentrations of 3.12, 6.25, 12.50, 25.00, 50.00, and 100.00 mg/L. **C** Microscopical images of organoids after treatment with different concentrations of nanoinhibitor. **D** AM/PI staining images of organoids after treatment with different concentrations of the nanoinhibitor. CC, Colorectal cancer. *P < 0.05, **P < 0.01, ***P < 0.001, and ****P < 0.0001. NS, not significant

Based on promising results in vitro and vivo, we further conducted research in organoids to evaluate the antitumor effect of the nanoinhibitor. The CRC organoid derived from two patients were treated with EGCG 37 °C, nanoinhibitor 37 °C, EGCG 43 °C, and nanoinhibitor 43 °C for 72 h. As shown in Fig. 8A, nanoinhibitor effectively reduced the HSP90 level in organoid, both under 37 °C and 43 °C treatment. Evaluated by the CCK-8 assay, nanoinhibitor exhibited evident cytotoxicity in 37 °C condition, and such effect could be strengthened significantly by combining with 43 °C heating (Fig. 8B). Under bright-field and AM/PI-stained fluorescence microscopy, the organoids showed morphological changes. The organoids, after treated with higher drug concentrations, were ruptured into small fragments, resulting in more deaths (Fig. 8C, D). These results suggested that the nanoinhibitor also inhibited HSP90 and showed considerable antitumor effect in patient-derived organoids.

### Biosafety evaluation of nanomedicine

**Fig. 9:**
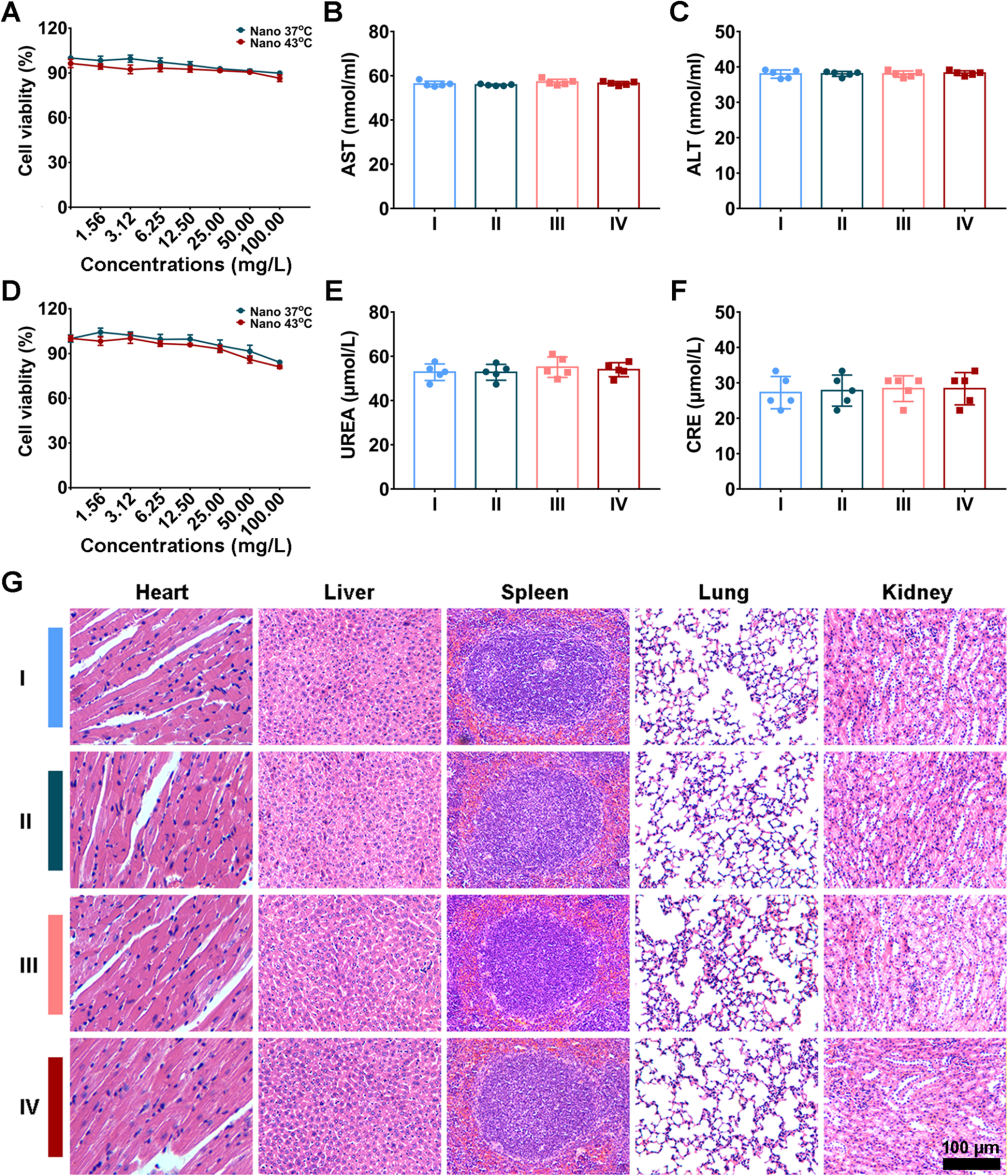
Biosafety evaluation of nanomedicine. **A** Cell viabilities of GSE1 cells treated with Nano 37 °C, Nano 43 °C for 24 h. **B**, **C** Biochemical markers of liver function (ALT, AST) of mice with different treatments. **D** Cell viabilities of HMrSV5 cells treated for 24 h. **E**, **F** Biochemical markers of kidney function (UREA, CRE) of mice with different treatments. **G** H&E staining images of main organs of mice with different treatments

Previous studies reported the HSP90α complex (consisting of different HSP and co-chaperones) from cancer cells revealed a 100-fold greater binding affinity with inhibitors than the homodimeric HSP90α from normal cells. Thus HSP90 inhibitors exert cytotoxicity on tumor cells with minimal damage to normal cells [17, 59]. In this study, the CCK-8 assay revealed no significant inhibition in the proliferation of normal human cell lines with the application of the nanoinhibitor for 24 h, even under heated condition (Fig. 9A, D). Similarly, normal cells exhibited more tolerance to Mn^2+^ and EGCG than tumor cells, probably because the less oxidative stress and HSP90 binding affinity in cancer cells (Fig. S17). For in vivo experiments, the liver (Fig. 9B, C) and renal function (Fig. 9E, F) related markers were in the normal range for the mice treated with nanoinhibitor-based HIPEC compared with control group. In addition, no significant morphological differences in main organs of mice were observed among different treatment groups and control group (Fig. 9G). The body weight showed little difference comparing to control group, which also suggested the nanoinhibitor has no obvious side effects (Fig. S18). These results together indicated that normal organs and tissues were more tolerant to this nanoinhibitor-based HIPEC therapy.

## Discussion

Heat has long been applied for tumor treatment. Tumors exposed to temperatures between 40 °C to 47 °C begin to lose viability, and show more frank necrosis under higher temperatures above 50 °C [60, 61]. However, heat alone may be insufficient to induce significant tumor cell death due to the non-uniform distribution of heat within the tumor tissue. The tumor cells surviving heat therapy will become heat-resistant even under increased temperature, duration, or frequency, causing tumor relapse and metastases. Thus, chemotherapy or radiotherapy is used as an adjuvant with heat for amplifying the antitumor efficacy, and the HIPEC is a successful example where heat treatment and chemotherapy are used.

Our work demonstrates the therapeutic potential of a novel engineered HSP90 inhibitor for HIPEC therapy. We designed a nanoinhibitor by specifically targeting the heat-resistance mechanisms of tumor cells at the molecular level. The nanoinhibitor showed significant tumor inhibition in HIPEC therapy by preferring to bind HSP90 in tumor cells rather than normal cells because of abundancy and high affinity in tumor cells [17]. The inhibition of HSP90 chaperone resulted in accumulation of unfolded/misfolded proteins, which were released into tumor microenvironment and served as an antigen reservoir for immune response [62]. In addition, the protein heat-denatured effect could further strengthen tumor immunogenicity. Tumor antigens were presented to APC cells and activated the immune response. Consistent with this notion, we found that tumor cells treated by the nanoinhibitor induced pyroptosis and maturation of DC, an effect further strengthened by heat. As previously reported, metalloimmunotherapy Mn^2+^ is a potent innate immune inducer, stimulating an IFN-γ response and cytokine generation locoregionally and through the circulation system [63, 64]. Emerging evidence also indicated heat enhances the synergistic effect of activated immune responses and reverses immunosuppression when combined with chemotherapy, immunotherapy, or any other therapy [65]. After the direct antiproliferation effect achieved by the nanoinhibitor, the second attack was accomplished by the immune response.

The nanoinhibitor showed good stability and tumor selectivity in terms of physicochemical properties. The nanoinhibitor prevented cell proliferation in multiple gastrointestinal tumor cell lines and inhibited the growth of tumor implanted in mice. To better mimic the clinical status of advanced colorectal tumor, we used organoids to assess the efficacy of the nanoinhibitor. Organoids are 3D in vitro cultures consisting of multiple organ-specific cells that can recapitulate the in vivo architecture functionality and genetic signature of their corresponding organs, serving as a promising platform for assessing efficacy of new therapies and predicting patient outcome in preclinical and clinical research [66]. The nanoinhibitor imposed a strong suppression of HSP90 in a patient-derived organoid. In addition, animal pharmacology studies indicated that the nanoinhibitor has high bioavailability and biosafety. The nanomedicine constructed from natural EGCG showed considerable advantages including improved efficacy, responsiveness to stimuli, and biocompatibility, with promising potential for future clinical translation.

However, there are several limitations in this work. Because EGCG exerted various biological and pharmacological activities, the antitumor mechanism of the nanoinhibitor at the transcriptional level still requires further study. Additionally, the accumulated unfolded/misfolded proteins due to HSP90 inhibition remain to be investigated. Grimmig et al. reported additional chemotherapy, such as 5-fluorocrail (5-FU), mitomycin C (MMC), and oxaliplatin (OXA), increased HSP90 expression when combined with hyperthermic perfusion [67]. We assumed if added to the current oxaliplatin and mitomycin C-based HIPEC or radiotherapy, the nanoinhibitor may have better antitumor effect.

## Conclusion

We report the design and characterization of a novel HSP90 nanoinhibitor for overcoming heat resistance and inducing tumor pyroptosis in colorectal tumor cells. The nanoinhibitor is formed by Mn^2+^ and the natural compound EGCG. After phagocytosed into tumor cells, the nanoinhibitor was able to inhibit heat resistance and induce synergistic cell‐killing effect with hyperthermic perfusion. Furthermore, pyroptosis induced by nanoinhibitor triggered immunogenic cell death and promoted maturation and antigen presentation of DCs. The efficacy and safety of nanoinhibitor-based HIPEC have also been confirmed in mice with peritoneal metastases of colorectal origin. The mechanism link goes as follow: increased oxidative stress added with stress resistance inhibition—pyroptosis—ICD—DCs maturation—immune activation, which further potentially promotes antitumor effect as an immunotherapy. In conclusion, the nanoinhibitor indicates a unique therapeutic strategy for multidisciplinary cancer treatment in the future.

## Supplementary Information

**Additional file 1:** **Fig. S1 to S18**.

## Data Availability

All data associated with this study are present in the paper and are obtained from the corresponding author.

## Abbreviations

HSP90: Heat shock protein 90
HSP70: Heat shock protein 70
HIPEC: Hyperthermic intraperitoneal chemotherapy
EGCG: Epigallocatechin gallate
PM: Peritoneal metastases
CRC: Colorectal cancer
ATP: Adenosine triphosphate
HSR: Heat shock response
FCN: Flash nanocomplexation
CIJM: Confined impingement jet mixer
CLSM: Laser scanning microscopy
CCK-8: Cell counting kit-8
AM/PI: Acetoxymethyl ester/propidium iodide
ROS: Reactive oxygen species
LDH: Lactate dehydrogenase
CRT: Calreticulin
HMGB1: High mobility group box 1
DC: Dendritic cell
ICD: Immunogenic cell death
IVIS: In vivo Imaging system
PCI: Peritoneal carcinomatosis index
ALT: Alanine aminotransferase
AST: Aspartate aminotransferase
UREA: Urea nitrogen
CRE: Creatinine

## References

[1] Sung H, Ferlay J, Siegel RL, Laversanne M, Soerjomataram I, Jemal A, Bray F (2021). Global Cancer Statistics 2020: GLOBOCAN Estimates of Incidence and Mortality Worldwide for 36 Cancers in 185 Countries. CA Cancer J Clin.

[2] Gamboa AC, Zaidi MY, Lee RM, Speegle S, Switchenko JM, Lipscomb J, Cloyd JM, Ahmed A, Grotz T, Leiting J, Fournier K, Lee AJ, Dineen S, Powers BD, Lowy AM, Kotha NV, Clarke C, Gamblin TC, Patel SH, Lee TC, Lambert L, Hendrix RJ, Abbott DE, Vande Walle K, Lafaro K, Lee B, Johnston FM, Greer J, Russell MC, Staley CA, Maithel SK (2020). Optimal Surveillance Frequency After CRS/HIPEC for Appendiceal and Colorectal Neoplasms: A Multi-institutional Analysis of the US HIPEC Collaborative. Ann Surg Oncol.

[3] Klaver CEL, Wisselink DD, Punt CJA, Snaebjornsson P, Crezee J, Aalbers AGJ, Brandt A, Bremers AJA, Burger JWA, Fabry HFJ, Ferenschild F, Festen S, van Grevenstein WMU, Hemmer PHJ, de Hingh IHJT, Kok NFM, Musters GD, Schoonderwoerd L, Tuynman JB, van de Ven AWH, van Westreenen HL, Wiezer MJ, Zimmerman DDE, van Zweeden AA, Dijkgraaf MGW, Tanis PJ, COLOPEC Collaborators Group (2019). Adjuvant hyperthermic intraperitoneal chemotherapy in patients with locally advanced colon cancer (COLOPEC): a multicentre, open-label, randomised trial. Lancet Gastroenterol Hepatol.

[4] Razenberg LG, van Gestel YR, Lemmens VE, de Hingh IH, Creemers GJ (2016). Bevacizumab in addition to palliative chemotherapy for patients with peritoneal carcinomatosis of colorectal origin: a nationwide population-based study. Clin Colorectal Cancer.

[5] Morris VK, Kennedy EB, Baxter NN, Benson AB, Cercek A, Cho M, Ciombor KK, Cremolini C, Davis A, Deming DA, Fakih MG, Gholami S, Hong TS, Jaiyesimi I, Klute K, Lieu C, Sanoff H, Strickler JH, White S, Willis JA, Eng C (2023). Treatment of metastatic colorectal cancer: ASCO Guideline. J Clin Oncol.

[6] Elias D, Bonnay M, Puizillou JM, Antoun S, Demirdjian S, El OA, Pignon JP, Drouard-Troalen L, Ouellet JF, Ducreux M (2002). Heated intra-operative intraperitoneal oxaliplatin after complete resection of peritoneal carcinomatosis: pharmacokinetics and tissue distribution. Ann Oncol.

[7] Van Stein RM, Aalbers AGJ, Sonke GS, van Driel WJ (2021). Hyperthermic intraperitoneal chemotherapy for ovarian and colorectal cancer: a review. JAMA Oncol.

[8] Francis P, Rowinsky E, Schneider J, Hakes T, Hoskins W, Markman M (1995). Phase I feasibility and pharmacologic study of weekly intraperitoneal paclitaxel: a Gynecologic Oncology Group pilot Study. J Clin Oncol.

[9] Hasovits C, Clarke S (2012). Pharmacokinetics and pharmacodynamics of intraperitoneal cancer chemotherapeutics. Clin Pharmacokinet.

[10] Sharma A, Özayral S, Caserto JS, Ten Cate R, Anders NM, Barnett JD, Kandala SK, Henderson E, Stewart J, Liapi E, Rudek MA, Franken NAP, Oei AL, Korangath P, Bunz F, Ivkov R (2019). Increased uptake of doxorubicin by cells undergoing heat stress does not explain its synergistic cytotoxicity with hyperthermia. Int J Hyperthermia.

[11] Palzer RJ, Heidelberger C (1973). Studies on the quantitative biology of hyperthermic killing of HeLa cells. Cancer Res.

[12] Hildebrandt B, Wust P, Ahlers O, Dieing A, Sreenivasa G, Kerner T, Felix R, Riess H (2002). The cellular and molecular basis of hyperthermia. Crit Rev Oncol Hematol.

[13] Pelz JO, Vetterlein M, Grimmig T, Kerscher AG, Moll E, Lazariotou M, Matthes N, Faber M, Germer CT, Waaga-Gasser AM, Gasser M (2013). Hyperthermic intraperitoneal chemotherapy in patients with peritoneal carcinomatosis: role of heat shock proteins and dissecting effects of hyperthermia. Ann Surg Oncol.

[14] Macario AJ, Conway de Macario E (2005). Sick chaperones, cellular stress, and disease. N Engl J Med..

[15] Saibil H (2013). Chaperone machines for protein folding, unfolding and disaggregation. Nat Rev Mol Cell Biol.

[16] Kasanga M, Liu L, Xue L, Song X (2018). Plasma heat shock protein 90-alpha have an advantage in diagnosis of colorectal cancer at early stage. Biomark Med.

[17] Trepel J, Mollapour M, Giaccone G, Neckers L (2010). Targeting the dynamic HSP90 complex in cancer. Nat Rev Cancer.

[18] Sidera K, Patsavoudi E (2014). HSP90 inhibitors: current development and potential in cancer therapy. Recent Pat Anticancer Drug Discov.

[19] Tu Y, Tian Y, Wu Y, Cui S (2018). Clinical significance of heat shock proteins in gastric cancer following hyperthermia stress: Indications for hyperthermic intraperitoneal chemoperfusion therapy. Oncol Lett.

[20] Moukarzel LA, Ferrando L, Dopeso H, Stylianou A, Basili T, Pareja F, Da Cruz PA, Zoppoli G, Abu-Rustum NR, Reis-Filho JS, Long Roche K, Tew WP, Chi DS, Sonoda Y, Zamarin D, Aghajanian C, O'Cearbhaill RE, Zivanovic O, Weigelt B (2022). Hyperthermic intraperitoneal chemotherapy (HIPEC) with carboplatin induces distinct transcriptomic changes in ovarian tumor and normal tissues. Gynecol Oncol.

[21] Taipale M, Jarosz DF, Lindquist S (2010). HSP90 at the hub of protein homeostasis: emerging mechanistic insights. Nat Rev Mol Cell Biol.

[22] Park HK, Yoon NG, Lee JE, Hu S, Yoon S, Kim SY, Hong JH, Nam D, Chae YC, Park JB, Kang BH (2020). Unleashing the full potential of Hsp90 inhibitors as cancer therapeutics through simultaneous inactivation of Hsp90, Grp94, and TRAP1. Exp Mol Med.

[23] Kamal A, Thao L, Sensintaffar J, Zhang L, Boehm MF, Fritz LC, Burrows FJ (2003). A high-affinity conformation of Hsp90 confers tumour selectivity on Hsp90 inhibitors. Nature.

[24] Mishra SJ, Liu W, Beebe K, Banerjee M, Kent CN, Munthali V, Koren J, Taylor JA, Neckers LM, Holzbeierlein J, Blagg BSJ (2021). The Development of Hsp90β-Selective Inhibitors to Overcome Detriments Associated with pan-Hsp90 Inhibition. J Med Chem.

[25] Maloney A, Clarke PA, Naaby-Hansen S, Stein R, Koopman JO, Akpan A, Yang A, Zvelebil M, Cramer R, Stimson L, Aherne W, Banerji U, Judson I, Sharp S, Powers M, deBilly E, Salmons J, Walton M, Burlingame A, Waterfield M, Workman P (2007). Gene and protein expression profiling of human ovarian cancer cells treated with the heat shock protein 90 inhibitor 17-allylamino-17-demethoxygeldanamycin. Cancer Res.

[26] Bhatia S, Spanier L, Bickel D, Dienstbier N, Woloschin V, Vogt M, Pols H, Lungerich B, Reiners J, Aghaallaei N, Diedrich D, Frieg B, Schliehe-Diecks J, Bopp B, Lang F, Gopalswamy M, Loschwitz J, Bajohgli B, Skokowa J, Borkhardt A, Hauer J, Hansen FK, Smits SHJ, Jose J, Gohlke H, Kurz T (2022). Development of a First-in-Class Small-Molecule Inhibitor of the C-Terminal Hsp90 Dimerization. ACS Cent Sci.

[27] Park JM, Kim YJ, Park S, Park M, Farrand L, Nguyen CT, Ann J, Nam G, Park HJ, Lee J, Kim JY, Seo JH (2020). A novel HSP90 inhibitor targeting the C-terminal domain attenuates trastuzumab resistance in HER2-positive breast cancer. Mol Cancer.

[28] Yang CS, Wang X, Lu G, Picinich SC (2009). Cancer prevention by tea: animal studies, molecular mechanisms and human relevance. Nat Rev Cancer.

[29] Almatroodi SA, Almatroudi A, Khan AA, Alhumaydhi FA, Alsahli MA, Rahmani AH (2020). Potential Therapeutic Targets of Epigallocatechin Gallate (EGCG), the Most Abundant Catechin in Green Tea, and Its Role in the Therapy of Various Types of Cancer. Molecules.

[30] Aggarwal V, Tuli HS, Tania M, Srivastava S, Ritzer EE, Pandey A, Aggarwal D, Barwal TS, Jain A, Kaur G, Sak K, Varol M, Bishayee A (2022). Molecular mechanisms of action of epigallocatechin gallate in cancer: Recent trends and advancement. Semin Cancer Biol.

[31] Tran PL, Kim SA, Choi HS, Yoon JH, Ahn SG (2010). Epigallocatechin-3-gallate suppresses the expression of HSP70 and HSP90 and exhibits anti-tumor activity in vitro and in vivo. BMC Cancer.

[32] Moses MA, Henry EC, Ricke WA, Gasiewicz TA (2015). The heat shock protein 90 inhibitor, (-)-epigallocatechin gallate, has anticancer activity in a novel human prostate cancer progression model. Cancer Prev Res.

[33] Chen D, Wan SB, Yang H, Yuan J, Chan TH, Dou QP (2011). EGCG, green tea polyphenols and their synthetic analogs and prodrugs for human cancer prevention and treatment. Adv Clin Chem.

[34] Chung JE, Tan S, Gao SJ, Yongvongsoontorn N, Kim SH, Lee JH, Choi HS, Yano H, Zhuo L, Kurisawa M, Ying JY (2014). Self-assembled micellar nanocomplexes comprising green tea catechin derivatives and protein drugs for cancer therapy. Nat Nanotechnol.

[35] Bae KH, Tan S, Yamashita A, Ang WX, Gao SJ, Wang S, Chung JE, Kurisawa M (2017). Hyaluronic acid-green tea catechin micellar nanocomplexes: Fail-safe cisplatin nanomedicine for the treatment of ovarian cancer without off-target toxicity. Biomaterials.

[36] Liang K, Chung JE, Gao SJ, Yongvongsoontorn N, Kurisawa M (2018). Highly Augmented Drug Loading and Stability of Micellar Nanocomplexes Composed of Doxorubicin and Poly(ethylene glycol)-Green Tea Catechin Conjugate for Cancer Therapy. Adv Mater.

[37] Trott O, Olson AJ (2010). AutoDock Vina: improving the speed and accuracy of docking with a new scoring function, efficient optimization, and multithreading. J Comput Chem.

[38] Lee K, Thwin AC, Nadel CM, Tse E, Gates SN, Gestwicki JE, Southworth DR (2021). The structure of an Hsp90-immunophilin complex reveals cochaperone recognition of the client maturation state. Mol Cell.

[39] Lu T, Chen F (2012). Multiwfn: a multifunctional wavefunction analyzer. J Comput Chem.

[40] Pettersen EF, Goddard TD, Huang CC, Meng EC, Couch GS, Croll TI, Morris JH, Ferrin TE (2021). UCSF ChimeraX: Structure visualization for researchers, educators, and developers. Protein Sci.

[41] Biovia DS. Discovery studio visualizer. San Diego, CA, USA 936; 2017. https://scholar.google.com/scholar_lookup?title=Discovery+studio+visualizer&author=D.+S.+Biovia&publication_year=2017&.

[42] Huang X, Qiu M, Wang T, Li B, Zhang S, Zhang T, Liu P, Wang Q, Qian ZR, Zhu C, Wu M, Zhao J (2022). Carrier-free multifunctional nanomedicine for intraperitoneal disseminated ovarian cancer therapy. J Nanobiotechnol.

[43] Ke X, Tang H, Mao HQ (2019). Effective encapsulation of curcumin in nanoparticles enabled by hydrogen bonding using flash nanocomplexation. Int J Pharm.

[44] Hu H, Yang C, Li M, Shao D, Mao HQ, Leong KW (2021). Flash Technology-Based Self-Assembly in Nanoformulation: From Fabrication to Biomedical Applications. Mater Today (Kidlington).

[45] Shi M, Ying DY, Hlaing MM, Ye JH, Sanguansri L, Augustin MA (2019). Development of broccoli by-products as carriers for delivering EGCG. Food Chem.

[46] Wang D, Kim D, Shin CH, Zhao Y, Park JS, Ryu M (2019). Evaluation of epigallocatechin gallate (EGCG) to remove Pb(II) using spectroscopic and quantum chemical calculation method. Environ Earth Sci.

[47] Li Y, Zhang T, Jiang Y, Lee HF, Schwartz SJ, Sun D (2009). (-)-Epigallocatechin-3-gallate inhibits Hsp90 function by impairing Hsp90 association with cochaperones in pancreatic cancer cell line Mia Paca-2. Mol Pharm.

[48] Yin Z, Henry EC, Gasiewicz TA (2009). (-)-Epigallocatechin-3-gallate is a novel Hsp90 inhibitor. Biochemistry.

[49] Schopf FH, Biebl MM, Buchner J (2017). The HSP90 chaperone machinery. Nat Rev Mol Cell Biol.

[50] Sun X, Zhang G, Du R, Xu R, Zhu D, Qian J, Bai G, Yang C, Zhang Z, Zhang X, Zou D, Wu Z (2019). A biodegradable MnSiO3@Fe3O4 nanoplatform for dual-mode magnetic resonance imaging guided combinatorial cancer therapy. Biomaterials.

[51] Pelicano H, Carney D, Huang P (2004). ROS stress in cancer cells and therapeutic implications. Drug Resist Updat.

[52] Yu L, Xu Y, Pu Z, Kang H, Li M, Sessler JL, Kim JS (2022). Photocatalytic Superoxide Radical Generator that Induces Pyroptosis in Cancer Cells. J Am Chem Soc.

[53] Shi J, Zhao Y, Wang K, Shi X, Wang Y, Huang H, Zhuang Y, Cai T, Wang F, Shao F (2015). Cleavage of GSDMD by inflammatory caspases determines pyroptotic cell death. Nature.

[54] Loveless R, Bloomquist R, Teng Y (2021). Pyroptosis at the forefront of anticancer immunity. J Exp Clin Cancer Res.

[55] Hsu SK, Li CY, Lin IL, Syue WJ, Chen YF, Cheng KC, Teng YN, Lin YH, Yen CH, Chiu CC (2021). Inflammation-related pyroptosis, a novel programmed cell death pathway, and its crosstalk with immune therapy in cancer treatment. Theranostics.

[56] Hou J, Hsu JM, Hung MC (2021). Molecular mechanisms and functions of pyroptosis in inflammation and antitumor immunity. Mol Cell.

[57] Lipscomb MF, Masten BJ (2002). Dendritic cells: immune regulators in health and disease. Physiol Rev.

[58] Klaver YL, Hendriks T, Lomme RM, Rutten HJ, Bleichrodt RP, de Hingh IH (2011). Hyperthermia and intraperitoneal chemotherapy for the treatment of peritoneal carcinomatosis: an experimental study. Ann Surg.

[59] Pearl LH, Prodromou C (2006). Structure and mechanism of the Hsp90 molecular chaperone machinery. Annu Rev Biochem.

[60] Wust P, Hildebrandt B, Sreenivasa G, Rau B, Gellermann J, Riess H, Felix R, Schlag PM (2002). Hyperthermia in combined treatment of cancer. Lancet Oncol.

[61] Roti Roti JL (2008). Cellular responses to hyperthermia (40–46 degrees C): cell killing and molecular events. Int J Hyperthermia.

[62] Graner MW (2016). HSP90 and immune modulation in cancer. Adv Cancer Res.

[63] Wang C, Guan Y, Lv M, Zhang R, Guo Z, Wei X, Du X, Yang J, Li T, Wan Y, Su X, Huang X, Jiang Z (2018). Manganese Increases the Sensitivity of the cGAS-STING Pathway for Double-Stranded DNA and Is Required for the Host Defense against DNA Viruses. Immunity.

[64] Sun X, Zhang Y, Li J, Park KS, Han K, Zhou X, Xu Y, Nam J, Xu J, Shi X, Wei L, Lei YL, Moon JJ (2021). Amplifying STING activation by cyclic dinucleotide-manganese particles for local and systemic cancer metalloimmunotherapy. Nat Nanotechnol.

[65] Chang M, Hou Z, Wang M, Li C, Lin J (2021). Recent advances in hyperthermia therapy-based synergistic immunotherapy. Adv Mater.

[66] Lau HCH, Kranenburg O, Xiao H, Yu J (2020). Organoid models of gastrointestinal cancers in basic and translational research. Nat Rev Gastroenterol Hepatol.

[67] Grimmig T, Moll EM, Kloos K, Thumm R, Moench R, Callies S, Kreckel J, Vetterlein M, Pelz J, Polat B, Tripathi S, Rehder R, Ribas CM, Chandraker A, Germer CT, Waaga-Gasser AM, Gasser M (2017). Upregulated heat shock proteins after hyperthermic chemotherapy point to induced cell survival mechanisms in affected tumor cells from peritoneal carcinomatosis. Cancer Growth Metastasis.

