# Supplementary material for: Metal-enriched HSP90 nanoinhibitor overcomes heat resistance in hyperthermic intraperitoneal chemotherapy used for peritoneal metastases
Source: Mol Cancer. 2023 Jun 14;22:95. doi: 10.1186/s12943-023-01790-2 (PMC10265871; doi:10.1186/s12943-023-01790-2)
Supplement: Supplementary file 1 — Additional file 1: Fig. S1 to S18. [file 12943_2023_1790_MOESM1_ESM.docx]

**Supplementary information**

**Metal-enriched HSP90 nanoinhibitor overcomes heat resistance in hyperthermic intraperitoneal chemotherapy used for peritoneal metastases**

Qiang Wang^a,b,c,†^, Peng Liu ^a,b,c,†^, Yingfei Wen^c^, Kuan Li ^a,b,c^, Bo Bi ^a,b,c^, Bin-bin Li^a,b^, Miaojuan Qiu^a,b^, Shiqiang Zhang^c^, You Li^c^, Jia Li^a,b^, Hengxing Chen^a,b^, Yuan Yin^d^, Leli Zeng ^a,b,c,*^, Changhua Zhang ^a,b,c,*^, Yulong He ^a,b,c,*^, Jing Zhao^a,c,*^


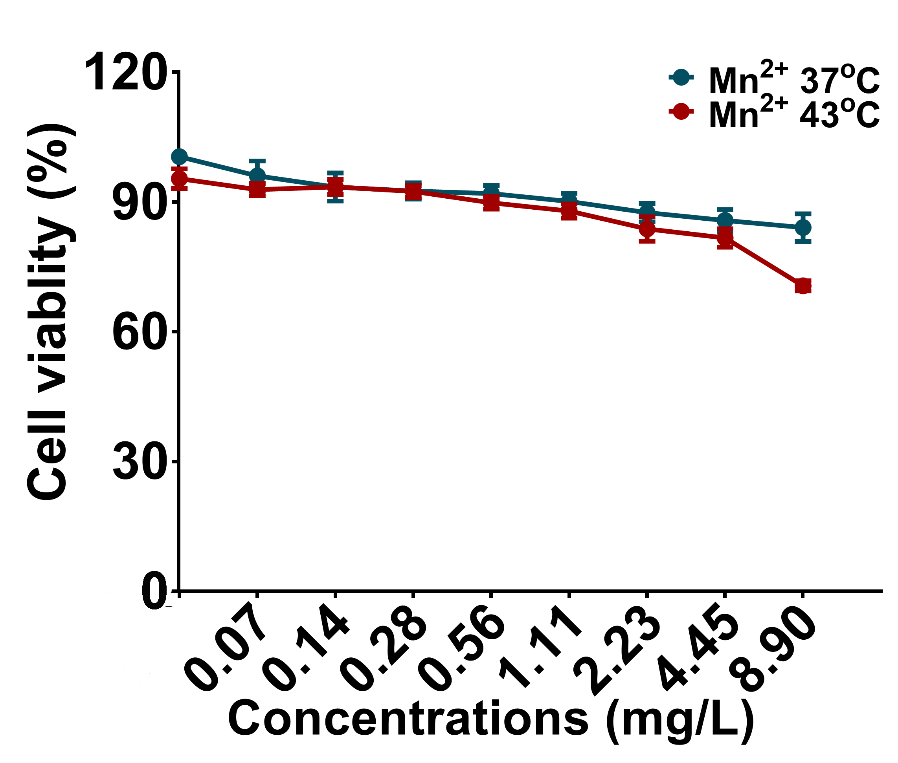


**Fig. S1** Cell viabilities of CT26 cells after treated with Mn^2+^ 37^o^C and Mn^2+^ 43^o^C for 24h.


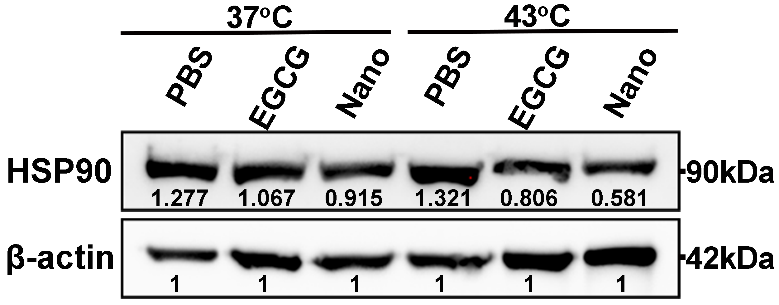


**Fig. S2** Western blot analysis of HSP90 level in HCT116 cells after different treatment.


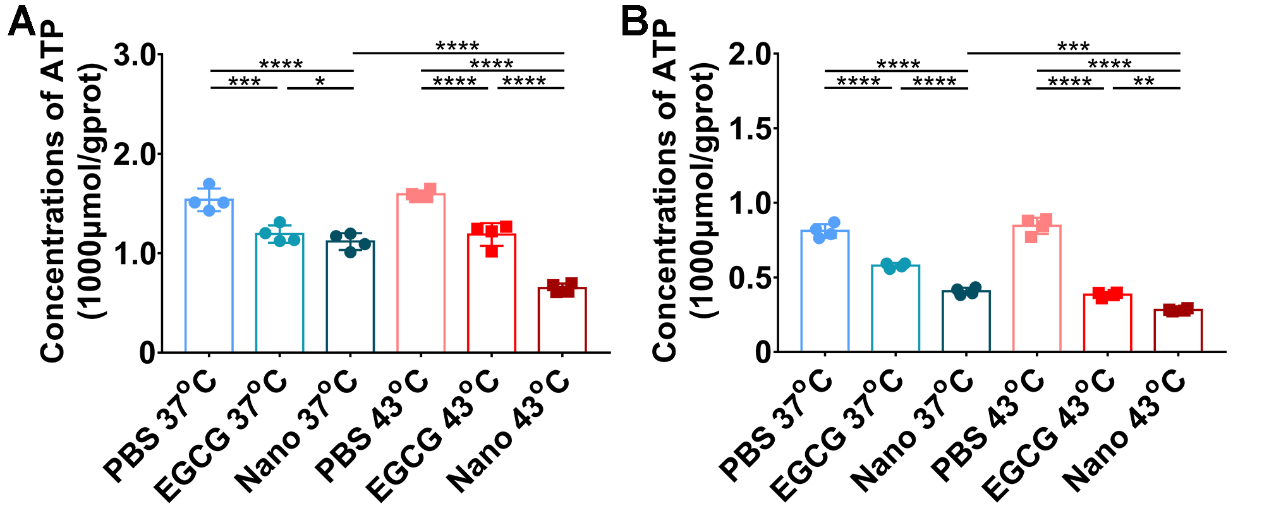
**Fig. S3** Intracellular ATP level of HCT116 (A) and CT26 (B) tumor cells respectively after different treatments. **p* < 0.05, ***p* < 0.01, ****p* < 0.001, and *****p* < 0.0001. Nano, nanoinhibitor.


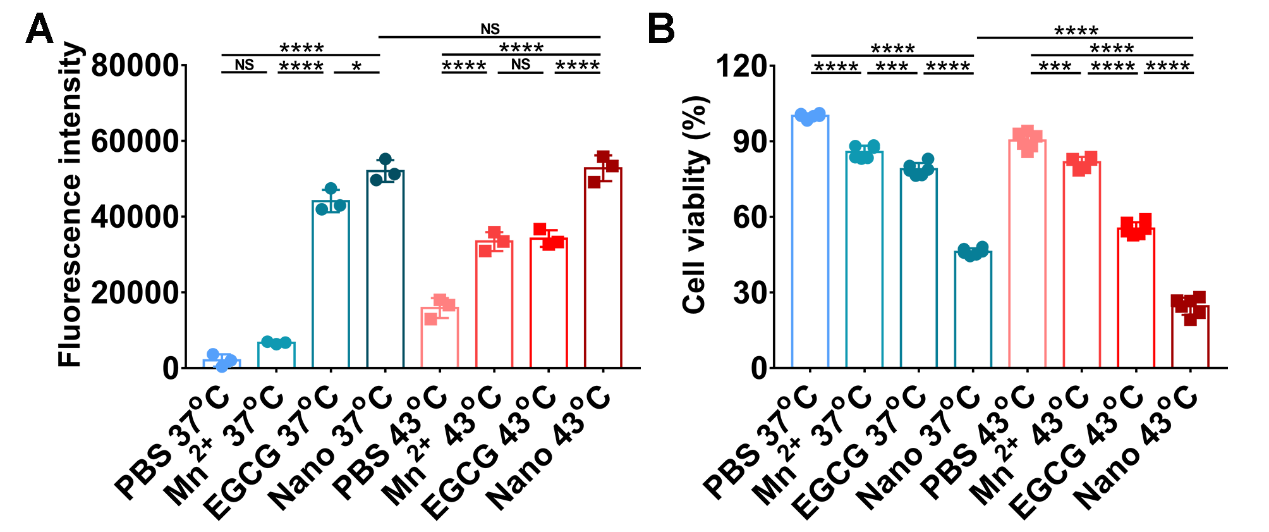


**Fig. S4** Fluorescence intensity of ROS levels. (A) Qualitative analysis of ROS levels produced in tumor cells after treatments with PBS 37^o^C, Mn^2+^ 37^o^C (with corresponding concentrations of 50 mg/L nanoinhibitor), EGCG (50 mg/L) 37^o^C, Nano (50 mg/L) 37^o^C, PBS 43^o^C, Mn^2+^ 43^o^C (with corresponding concentrations of 50 mg/L nanoinhibitor), EGCG (50 mg/L) 43^o^C, and Nano (50 mg/L) 43^o^C. (B) Cell viabilities of tumor cells after treatments with drug concentration of 50 mg/L. NS, not significant. **p* < 0.05, ****p* < 0.001, and *****p* < 0.0001. NS, not significant.


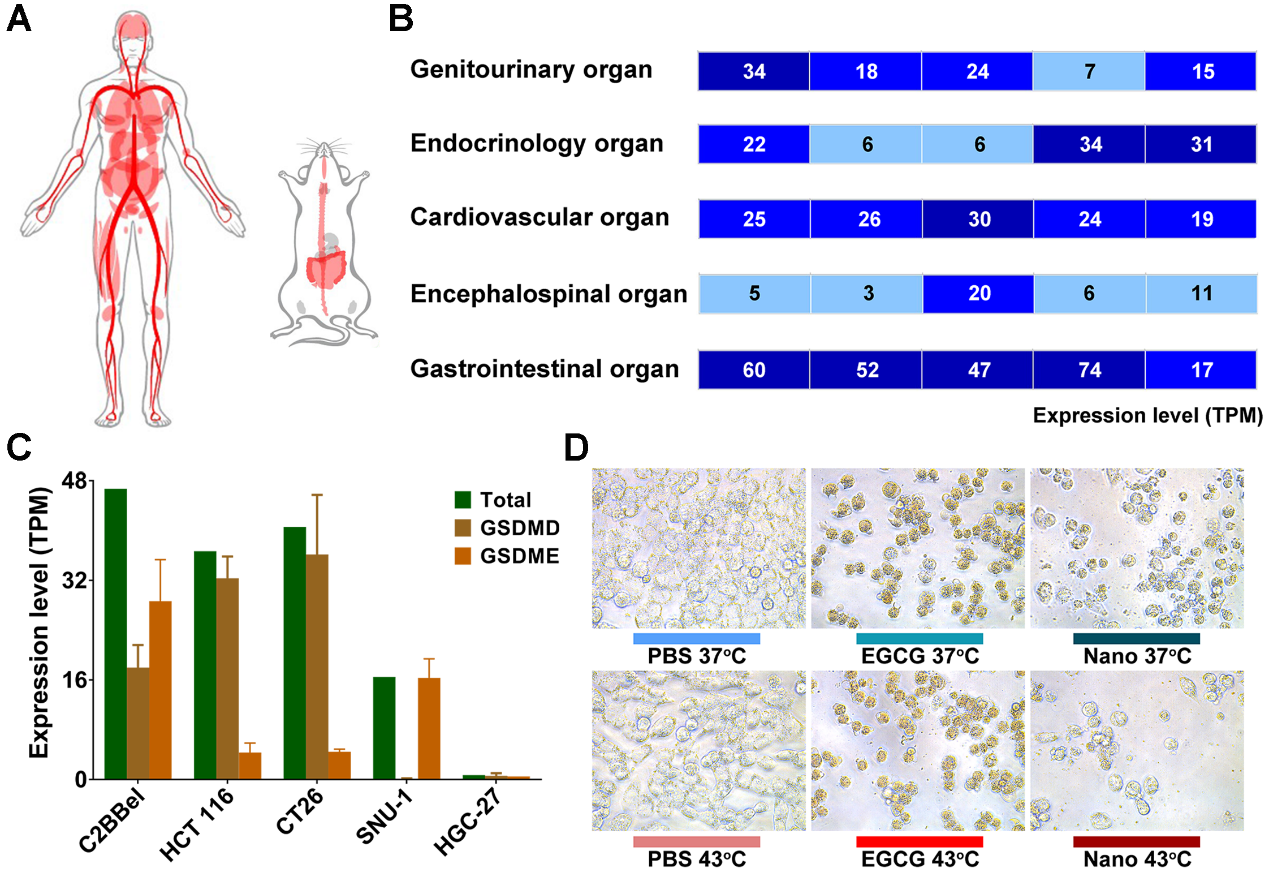
**Fig. S5** Pyroptosis of cells and organs. (A) Schematic illustration showing distribution of gasdermin protein expression in human and mouse (cited from the *Expression Atlas*). (B, C) Expression levels of gasdermins in different organs and cell lines. (D) Morphological changes of HGC-27 tumor cells (gasdermin-underexpressed) after different treatments.


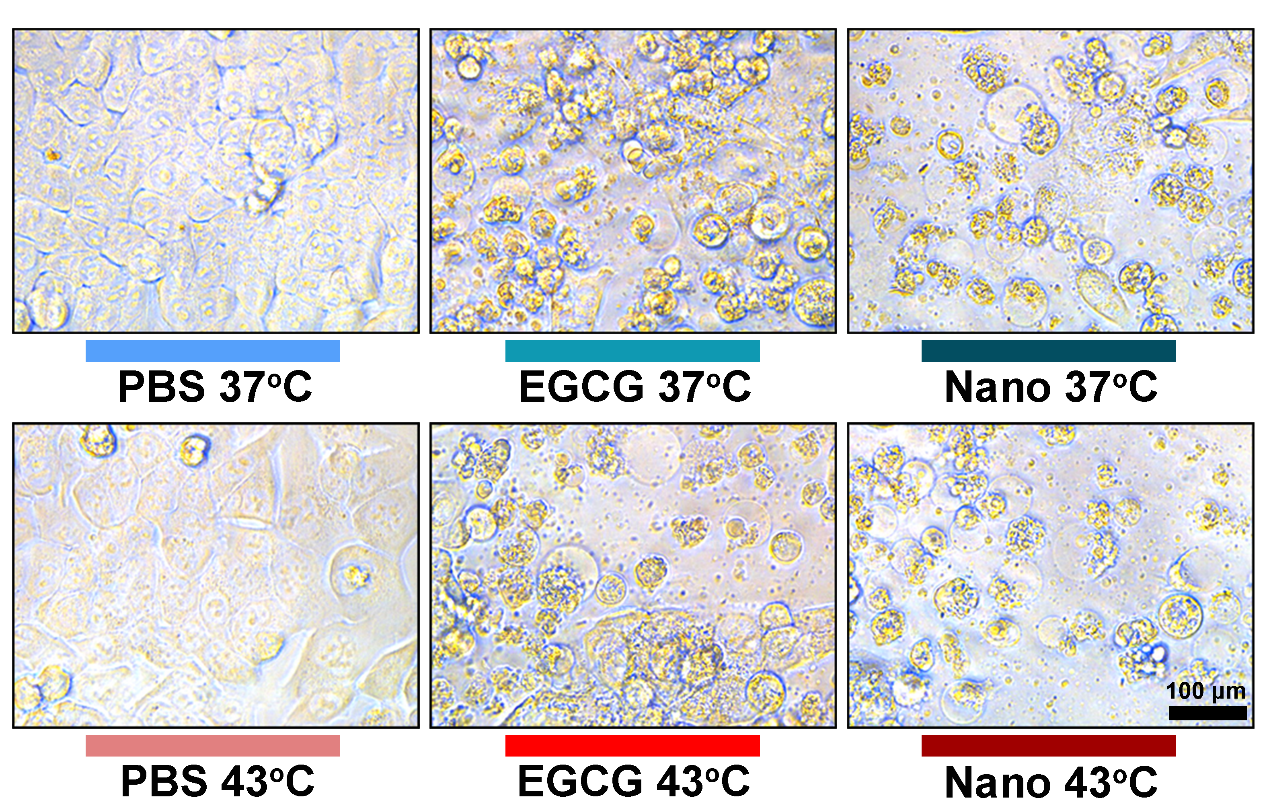
**Fig. S6** Brightfield images of pyroptosis induced by different treatments in HCT116 cell lines.


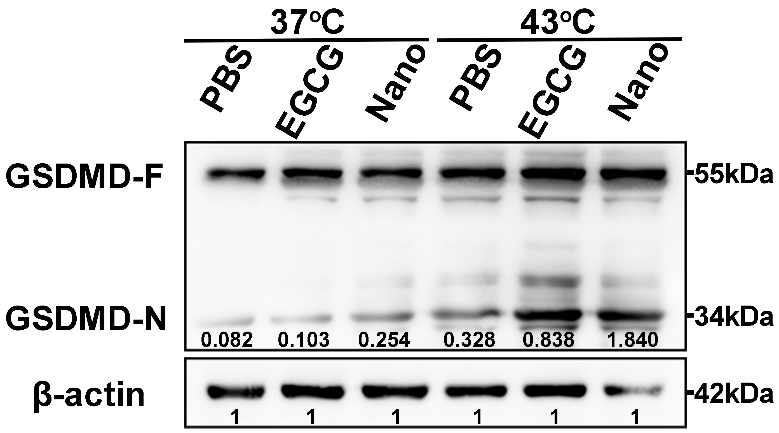


**Fig. S7** Western blot analysis of pyroptosis-related proteins in HCT116 cells after different treatment.


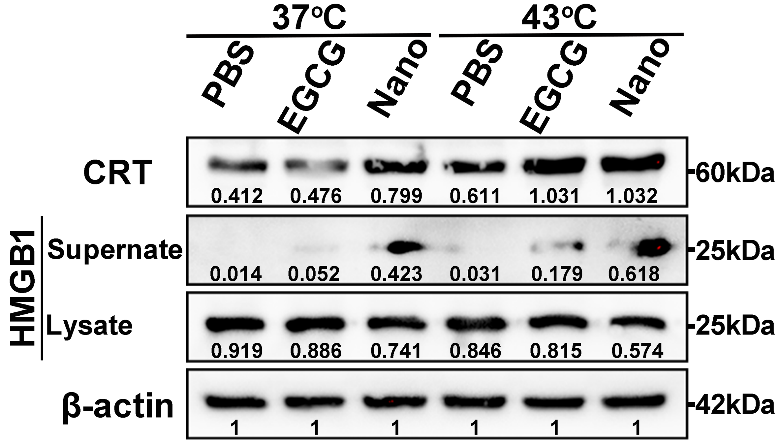


**Fig. S8** Immunogenic cell death induced by nanoinhibitor. Western blot analysis of expression of CRT and HMGB1 proteins from lysate and supernate.


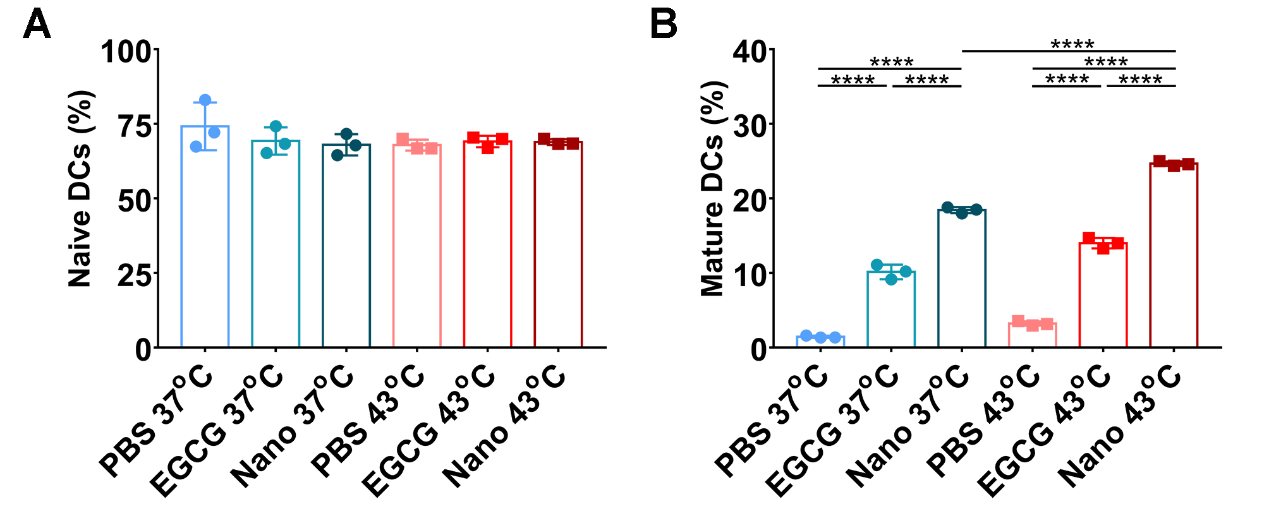
**Fig. S9** Changes of DCs number incubating tumor cells after different treatments. (A) Naive DCs from bone marrow in different groups. (B) Mature DCs induced by antigens from treated tumor cells. *****p* < 0.0001.


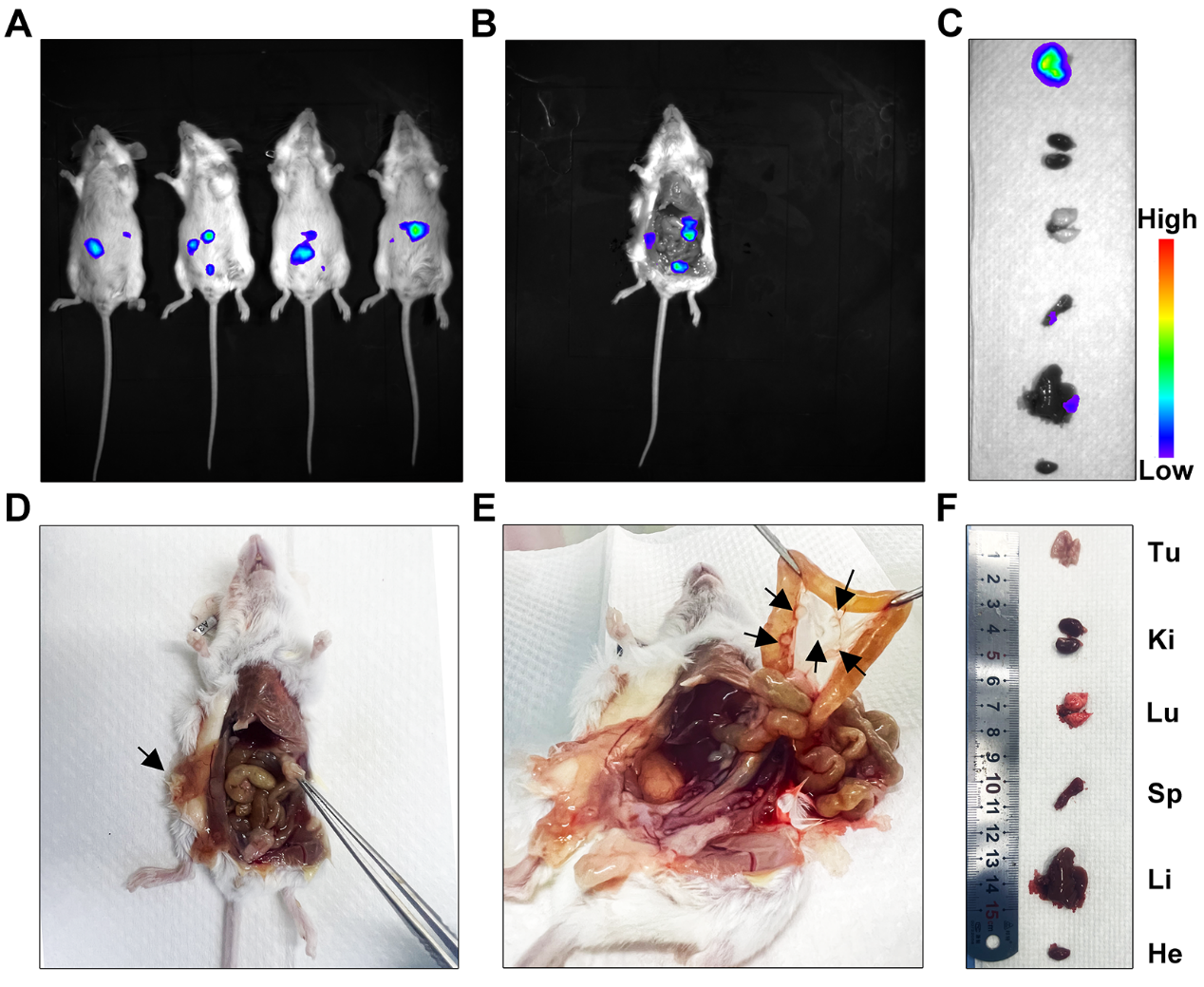
**Fig. S10** Establishment of colon cancer intraperitoneal metastasis model in Balb/c mouse. (A, B) Bioluminescence images of CT26 cells intraperitoneal metastasis in female Balb/c mouse at day 5. (C) Bioluminescence images of tumors and main organs of tumor-bearing mouse. (D, E) Representative images of tumors in intraperitoneal cavity. (F) Representative images of tumors and main organs of mouse.


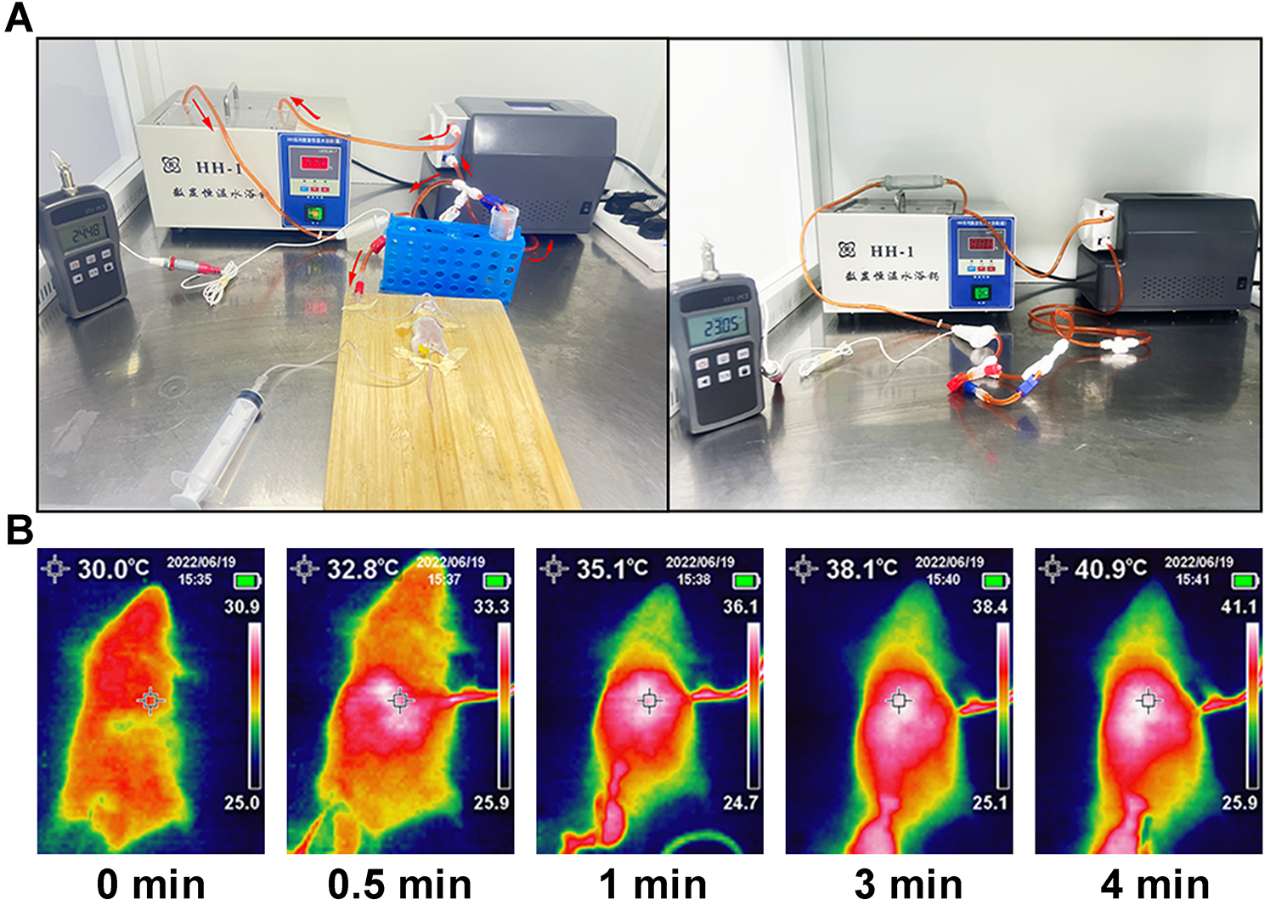
**Fig. S11** Establishment of hyperthermic intraperitoneal chemotherapy in mouse. (A) Images of equipments and procedures of hyperthermic intraperitoneal perfusion. (B) Temperatures changes as the time of intraperitoneal perfusion increased and maintained at higher than 41℃ but less than 43℃.


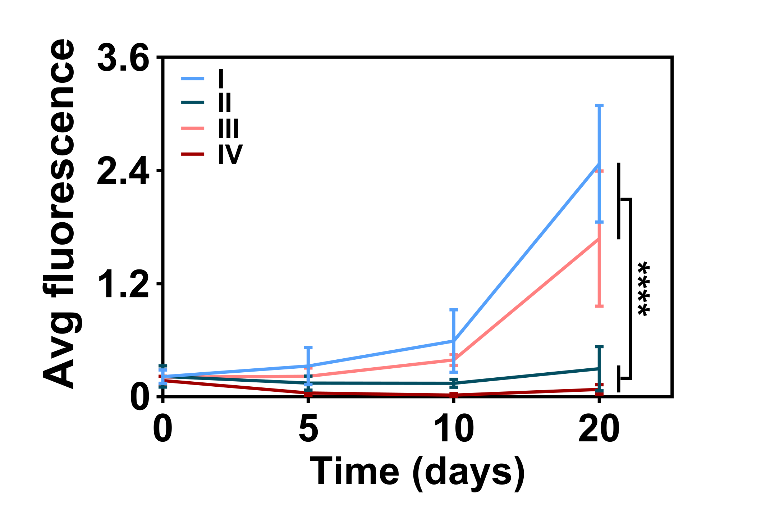


**Fig. S12** Average fluorescence intensity of mice *in vivo* treated with PBS, Nano 37^o^C, PBS 43^o^C, Nano 43^o^C. *****p* < 0.0001. Avg, Average.


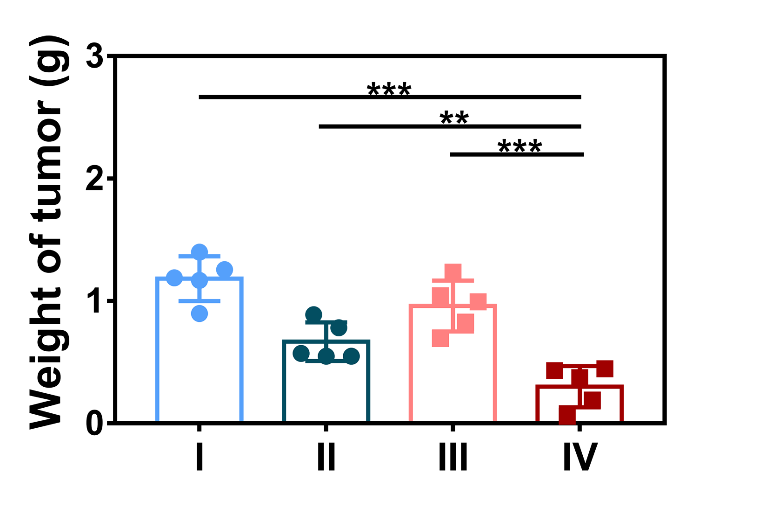


**Fig. S13** The weight of tumors from mice received different treatments. ***p* < 0.01. ****p* < 0.001.


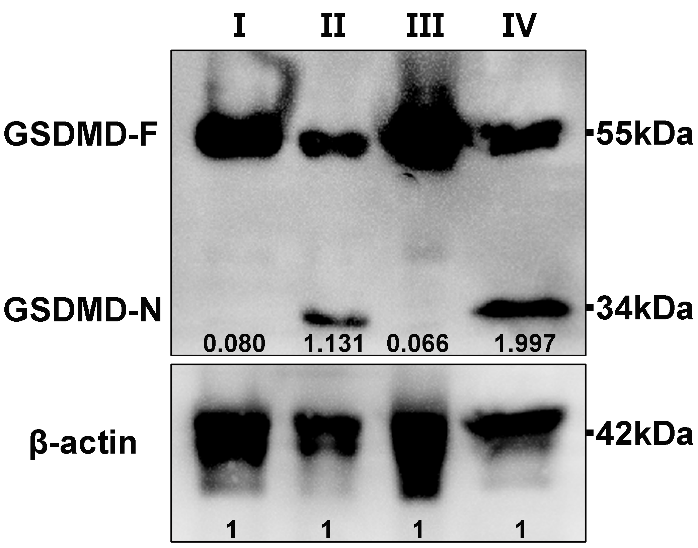


**Fig. S14** Western blot analysis of GSDMD level of tumor tissue in different treatment groups.

**
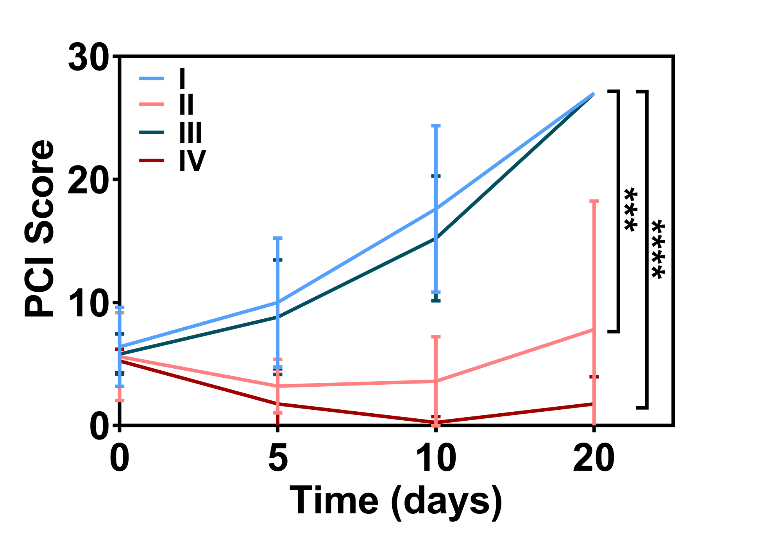
**

**Fig. S15** Average PCI score of mice with different treatments. ****p* < 0.001. *****p* < 0.0001.


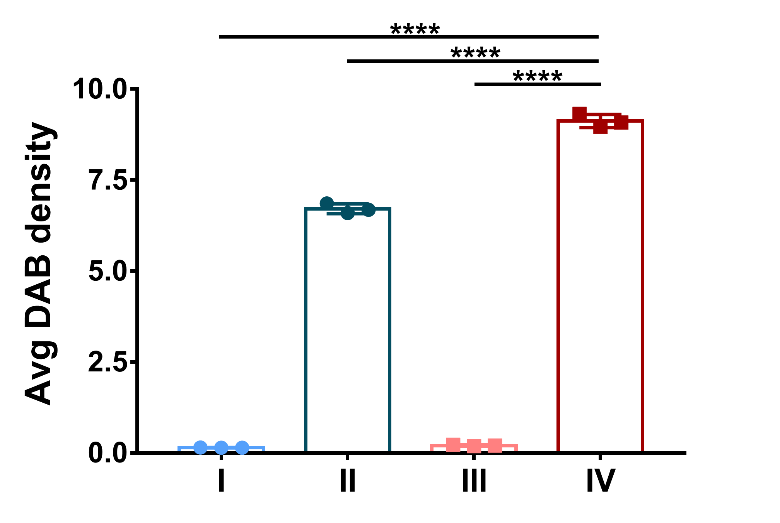


**Fig. S16** Average DAB density of CD3 antibody of tumor slices in different groups. ****p < 0.0001.


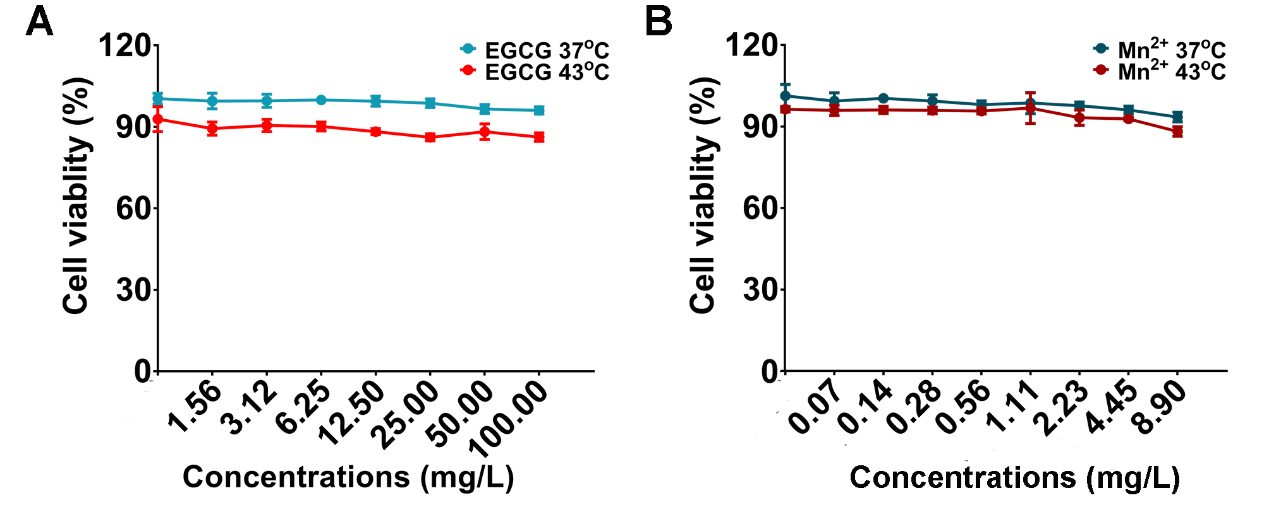


**Fig. S17** (A) Cell viabilities of HMrSV5 cells treated with EGCG 37^o^C, EGCG 43^o^C for 24 h. (B) Cell viabilities of HMrSV5 cells treated with Mn^2+^ 37^o^C, Mn^2+^ 43^o^C with corresponding concentrations of nanoinhibitor for 24 h.


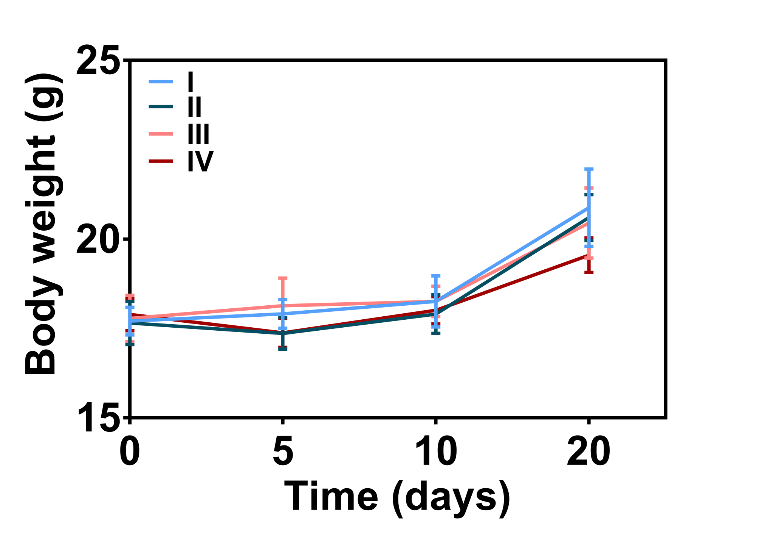


**Fig. S18** Body weight of mice with different treatments.
